# Comparative Study of Injury Models for Studying Muscle Regeneration in Mice

**DOI:** 10.1371/journal.pone.0147198

**Published:** 2016-01-25

**Authors:** David Hardy, Aurore Besnard, Mathilde Latil, Grégory Jouvion, David Briand, Cédric Thépenier, Quentin Pascal, Aurélie Guguin, Barbara Gayraud-Morel, Jean-Marc Cavaillon, Shahragim Tajbakhsh, Pierre Rocheteau, Fabrice Chrétien

**Affiliations:** 1 Institut Pasteur, Human histopathology and animal models Unit, Infection and Epidemiology Department, Paris, France; 2 Paris Est University, Créteil, France; 3 Paris Descartes University, Sorbonne Paris Cité, Paris France; 4 IRBA, Unité Interactions Hôte-Agents Pathogènes, Institut de Recherche Biomédicale des Armées, Brétigny-sur-Orge, France; 5 Inserm, U955, Plateforme de Cytométrie en Flux, Créteil, France; 6 Institut Pasteur, Stem Cells & Development Unit, Department of Developmental & Stem Cell Biology, Paris, France; 7 Institut Pasteur, Cytokines and Inflammation Unit, Infection and Epidemiology Department, Paris, France; 8 Centre Hospitalier Sainte Anne, Laboratoire de Neuropathologie, Paris, France; University of Louisville School of Medicine, UNITED STATES

## Abstract

**Background:**

A longstanding goal in regenerative medicine is to reconstitute functional tissus or organs after injury or disease. Attention has focused on the identification and relative contribution of tissue specific stem cells to the regeneration process. Relatively little is known about how the physiological process is regulated by other tissue constituents. Numerous injury models are used to investigate tissue regeneration, however, these models are often poorly understood. Specifically, for skeletal muscle regeneration several models are reported in the literature, yet the relative impact on muscle physiology and the distinct cells types have not been extensively characterised.

**Methods:**

We have used transgenic *Tg*:*Pax7nGFP* and *Flk1*^*GFP/+*^ mouse models to respectively count the number of muscle stem (satellite) cells (SC) and number/shape of vessels by confocal microscopy. We performed histological and immunostainings to assess the differences in the key regeneration steps. Infiltration of immune cells, chemokines and cytokines production was assessed *in vivo* by Luminex^®^.

**Results:**

We compared the 4 most commonly used injury models *i*.*e*. freeze injury (FI), barium chloride (BaCl_2_), notexin (NTX) and cardiotoxin (CTX). The FI was the most damaging. In this model, up to 96% of the SCs are destroyed with their surrounding environment (basal lamina and vasculature) leaving a “dead zone” devoid of viable cells. The regeneration process itself is fulfilled in all 4 models with virtually no fibrosis 28 days post-injury, except in the FI model. Inflammatory cells return to basal levels in the CTX, BaCl_2_ but still significantly high 1-month post-injury in the FI and NTX models. Interestingly the number of SC returned to normal only in the FI, 1-month post-injury, with SCs that are still cycling up to 3-months after the induction of the injury in the other models.

**Conclusions:**

Our studies show that the nature of the injury model should be chosen carefully depending on the experimental design and desired outcome. Although in all models the muscle regenerates completely, the trajectories of the regenerative process vary considerably. Furthermore, we show that histological parameters are not wholly sufficient to declare that regeneration is complete as molecular alterations (*e*.*g*. cycling SCs, cytokines) could have a major persistent impact.

## Introduction

Skeletal muscles represent up to 40% of the total body mass and are comprised largely of differentiated myofibres that have irreversibly left the cell cycle. Following injury of adult muscle, resident stem (satellite) cells (SCs) [1], which are mostly quiescent, re-enter the cell cycle and generate myoblasts that will participate in myofibre reconstitution or repair. The efficient reconstitution of functional muscle requires the coordinated action of other cell types including macrophages, fibro-adipogenic precursors, interstitial connective tissue and endothelial cells for blood vessel formation. This tissue regenerates *ad integrum* within a relatively short period (21 days in young adult mice) [2–4]. A first wave of inflammatory macrophages (MPs) stimulates myogenic cell proliferation whereas a second wave of antiinflammatory MPs promotes muscle differentiation [5]. Considerable crosstalk takes place between endothelial, fibro-adipogenic and myogenic cells to coordinate angiogenesis, connective tissue formation and remodelling and myogenesis [5–7].

Although muscle regeneration is highly efficient, this process can be compromised in several pathological conditions, during diseases such as myopathies, following trauma or infection. In human, this can result in severe handicap, organ failure even death. Understanding the mechanisms of tissue homeostasis, maintenance, and regeneration is crucial for devising innovative therapeutic strategies. In this context, the use of reproducible and controlled experimental models of muscle injury is essential. Currently, the most commonly used models for provoking muscle injury and repair include myotoxic agents (notexin, cardiotoxin), chemicals (barium chloride), and physical procedures (freeze injury, irradiation, crush, denervation and transplantation). It is generally believed that these muscle injury models begin with a phase of severe tissue necrosis followed by muscle regeneration and finally an *ad integrum* restitution of the tissue [2].

Skeletal muscle regeneration has emerged as a major paradigm to investigate the role of stem and stromal cells following tissue damage. However, different injury protocols are often indiscriminately employed in different laboratories. Due to their diverse variety and intensity, it is reasonable to assume that the regeneration process would be altered depending on the extent of imbalance in the response of stem and stromal cells. For example, we previously showed that freeze injury exposed a muscle regeneration phenotype in *Myf5* null mice, whereas this phenotype was masked following cardiotoxin injury [8]. Therefore, different injury protocols can differentially impact on cell types in the tissue thereby influencing profoundly the outcome on the regeneration process. Hence, it is critical to develop an understanding of tissue destruction mechanisms and how different cell types in the tissue respond under these conditions. This is a prerequisite for selecting the appropriate model for research on tissue regeneration in the context of disease or for cell therapies of skeletal muscle. For example, in recently reported studies, different outcomes were reported for the action of GDF11 as a rejuvenation factor during ageing [9,10]. Given that different injury models were used in those studies, it is possible that some of the differences are related to the injury model of choice [11] The development of standardized protocols should help (i) reduce the number of animals used in experimentation in accordance to ethical guidelines for animal well fare, (ii) homogenise data between different research teams working on muscle regeneration, and (iii) increase comparability between experiments and clarify differences when examining disease or mutant animal models.

The aim of the present study is to systematically compare the four most commonly used models: mechanical injury (freeze injury), myotoxins (notexin and cardiotoxin) and chemical agent (BaCl_2_). We used multiple approaches to describe alterations in tissue organisation, cellular and molecular landmarks and focused on satellite cells, vascular network, inflammation and connective tissue. This study revealed that despite similar initial necrosis and complete regeneration one month post-injury, significant differences could be detected between the different injury models, thereby permitting the selective use of distinct models in different contexts.

## Material and Methods

### Ethics

All mice were housed in a level 2 biosafety animal facility, and received food and water *ad libitum*. Prior to manipulations, animals (injured or controls) were anaesthetized using intraperitoneal injection of 100μl of Ketamine^®^ and Xylazine^®^ (respectively 80 mg/kg and 10 mg/kg), in Phosphate Buffer Saline (PBS).

This study was conducted in accordance with French and EU guidelines for animal care [French regulation n° 2013–118 of 02/01/2013 that transposes directive 2010/63/EU of 09/22/2010 on the protection of animals used for scientific purposes]. Protocols were approved by the Institut Pasteur Animal Experimentation Ethics Committee (n° 01332.01). In brief, mice were monitored every day (by the coordinator of the study, under the supervision of a veterinary pathologist) and killed by CO_2_ asphyxiation (5 L/min) to induce rapid unconsciousness with minimal distress to the animals. Our experiments had a limited impact on the mouse living condition. Our endpoints were: a weight loss of 20% or more, lethargy, piloerection, palpebral occlusion, and tremor.

### Mice

Most experiments were conducted with C57BL6/J mice (Charles River Laboratory, L’arbresle, France) or in genetically engineered mice backcrossed on a C57BL6/J background (at least 10 backcrosses).

*Tg*:*Pax7nGFP* mice were described previously [12]. In these animals all satellite cells express the Green Fluorescent Protein (GFP), allowing cell counting and prospective cell sorting.

*Flk-1*^*GFP/+*^ mice were used for endothelial cell morphological studies (kindly provided by A. Medvinsky, Institute for Stem Cell Research, University of Edinburgh, UK). GFP is targeted to the vascular endothelial growth factor (VEGF) receptor-2 gene locus [13].

Animals were used between 6 to 10 weeks after birth and at least five animals (n = 5) were used per condition and per time point.

### Muscle injury procedures

#### Freeze Injury

After skin incision and muscle exposition, *Tibialis anterior* (TA) muscles were frozen with three consecutive cycles of freeze-thawing by applying for 15 seconds a liquid nitrogen cooled metallic rod (S1A–S1E Fig). The skin was then sutured with 4.0 suture string and mice were kept at 37°C on a heating pad for 2 h.

#### Myotoxin injury

Cardiotoxin (a kinase C inhibitor) and (a phospholipase A2), extracted from snake venoms, are the most commonly used myotoxins. To produce experimental muscle injury, an intramuscular (IM) injection of 25 μl or 50 μl of cardiotoxin (10 μmol in 0.9% of PBS, Latoxan^®^, France) or 10 μl or 50 μl of notexin (12.5 μmol in 0.9% of PBS, Latoxan^®^, France), was injected with a 30 Gauge Hamilton microsyringe in the TA. To limit variability between toxin batches, 25 (25mg for CTX and 12.5mg for NTX) batches were reconstituted, pooled, aliquoted and stored at -20°C to standardize experiments.

#### Chemical injury

Chemical injury was carried out using IM injection of 10 μl or 50 μl of barium chloride (1.2% in sterile demineralized water) (Sigma Aldrich, MO, USA) in the TA, as previously described for myotoxins.

#### Re-injury

This model consists in two successive muscle lesions to study satellite cell pool maintenance or depletion after more than one round of regeneration. Practically, two lesions were carried out 28 days apart; the time elapsed between the two injuries allowing the regeneration of the muscle. As such, first injury, recovery for 28 days, re-injury, then recovery for 28 days followed by mouse sacrifice.

### Tissue preparation and histological analysis

For histopathological analysis, right and left TA muscles were collected and snap-frozen in liquid nitrogen-cooled isopentane. Six different levels of 7 μm-thick sections were cut and stained with hematoxylin-eosin (HE) to describe histological alterations, Von Kossa to evaluate calcium deposition, and Sirius red to visualise collagen deposits.

To preserve GFP fluorescence for the study of blood vessel 3D organisation, whole TA muscles were fixed in 10% neutral buffered formalin for 2 h, then cryopreserved in 40% sucrose overnight at 4°C before freezing in OCT (Tissue-Tek^®^, Sakura^®^ Finetek, CA, USA) in small cryomolds. Serial cryosections (7 μm or 100 μm-thick sections for 2D and 3D analysis, respectively) were performed.

Images were captured on a Nikon Eclipse E800 microscope with Nikon ACT-1 software and DXM1200 camera for bright field. Fluorescence images and 3D reconstructions were performed with Leica^®^ TCS SPE DM 2500 and LAS AF software (Leica^®^, Germany).

### Immunohistochemistry

Tissues were rehydrated in PBS, saturated with 20% goat serum (Sigma Aldrich, MO, USA) and permeabilised with 0.5% Triton X100 (Sigma Aldrich, MO, USA). Primary antibodies (Table 1) were incubated overnight at 4°C. Secondary antibodies were incubated 1 h at 37°C. Sections were counterstained with Hoechst 33342 (Life technologies^®^, CA, USA) for 5 min in PBS with 10 mg/ml, H3570 (Invitrogen, CA, USA). In case of chromogenic signal, after secondary biotinylated antibody incubation, endogenous peroxidases were blocked with 20% ethanol, 3% H_2_0_2_ in PBS. Amersham Streptavidin-peroxidase 1,6 μg/ml (GE Healthcare^®^, United Kingdom) was incubated for 30 min at 37°C and finally revealed using AEC system according to manufacturer procedure (Vector laboratories^®^, CA, USA).

**Table 1:** Summary and references of the primary antibodies used.

Antigen	Recongnized population	Host	Final used concentration	Providers references
**Ly-6C**	Granulocytes / Myeloid cells	Rat	0.5 μg/ml	Caltag LabRM3030
**F4/80**	Macrophages	Rat	4 μg/ml	Caltag LabMF48000
**CD45R (B220)**	B-cells	Rat	5 μg/ml	Caltag LabRM2600
**CD3**	T-cells	Rabbit	12 μg/ml	Dako A0452
**CD31**	Endothelial cells	Rat	15 μg/ml	BD Pharmingen 550274
**Pax7**	Satellite cells	Mouse	4 μg/ml	DSHB
**Laminin**	Basal lamina	Rabbit	0.7 μg/ml	Sigma L9393

### Satellite cell counting

To investigate satellite cell counts in one single isolated TA muscle we used transgenic *Tg*:*Pax7nGFP* mouse allowing the prospective selection by cytometry (FACS) and cell counting (S2A Fig). This method was validated by correlating the SC count obtained by FACS and detected on histological sections after Pax7 immunostaining. We also validated our protocol by comparing the consistency and reproducibility of the cell count throughout experiments (S2B Fig) Methods for muscle dissection and SC extraction were previously described [14].

### Cell proliferation

To study SC proliferation, we investigated the incorporation of the thymidine analog: 5-ethynyl-2’-deoxyuridine (EdU) (Invitrogen^®^, Carlsbad, CA, USA). Mice were injected intraperitoneally one-month post-injury with a solution (50mg/kg) of EdU 24 h and 4 h before the sacrifice. EdU staining was revealed according the manufacturer guidelines. Cell proliferation was also assessed by KI67 immunostaining.

### Multiplex cytokine and chemokine analysis

Snap frozen TA samples (n = 4 per condition) were thawed, lysed, and supernatant was processed for Luminex^®^ multiple cytokine and chemokine analysis (BioRad^®^, CA, USA) ProTM Mouse Cytokine Standard 23-Plex, Group I and Standard 9-Plex, Group II: FGF Basic, IL-15, IL-18, LIF, M-CSF, MIG, MIP-2, PDGF-BB, VEGF. Results were normalised on frozen muscle sample total weight.

### Statistical Analysis

Data are expressed as mean±SEM, unless otherwise indicated. Statistical analysis was performed using GraphPad software (Prism, CA, USA) with Mann-Whitney; p≤0.05 was considered statistically significant. p-values indicated on graphs are < 0.05 (*); < 0.001(**); < 0.0001 (***); no star or ns, statistically non significant.

### Morphometric analysis

Two-dimension analysis was performed. Using ImageJ (NIH, MA, USA) and NIS-Element (Nikon) softwares, we evaluated distribution of muscle fibre diameter and their number, and capillary count around each myofibre. At least 100 randomly selected fibres were considered for each muscle.

Three-dimensional analysis was performed to evaluate organisation of the vascular network. For each muscle, ten images were collected at 4 μm intervals to create a stack in the z axis. 3D reconstruction of this z-stack image was performed using 80 to 150 μm-thick frozen sections. Analysis was carried out using using ImageJ (NIH, MA, USA).

## Results

**Fig 1:**
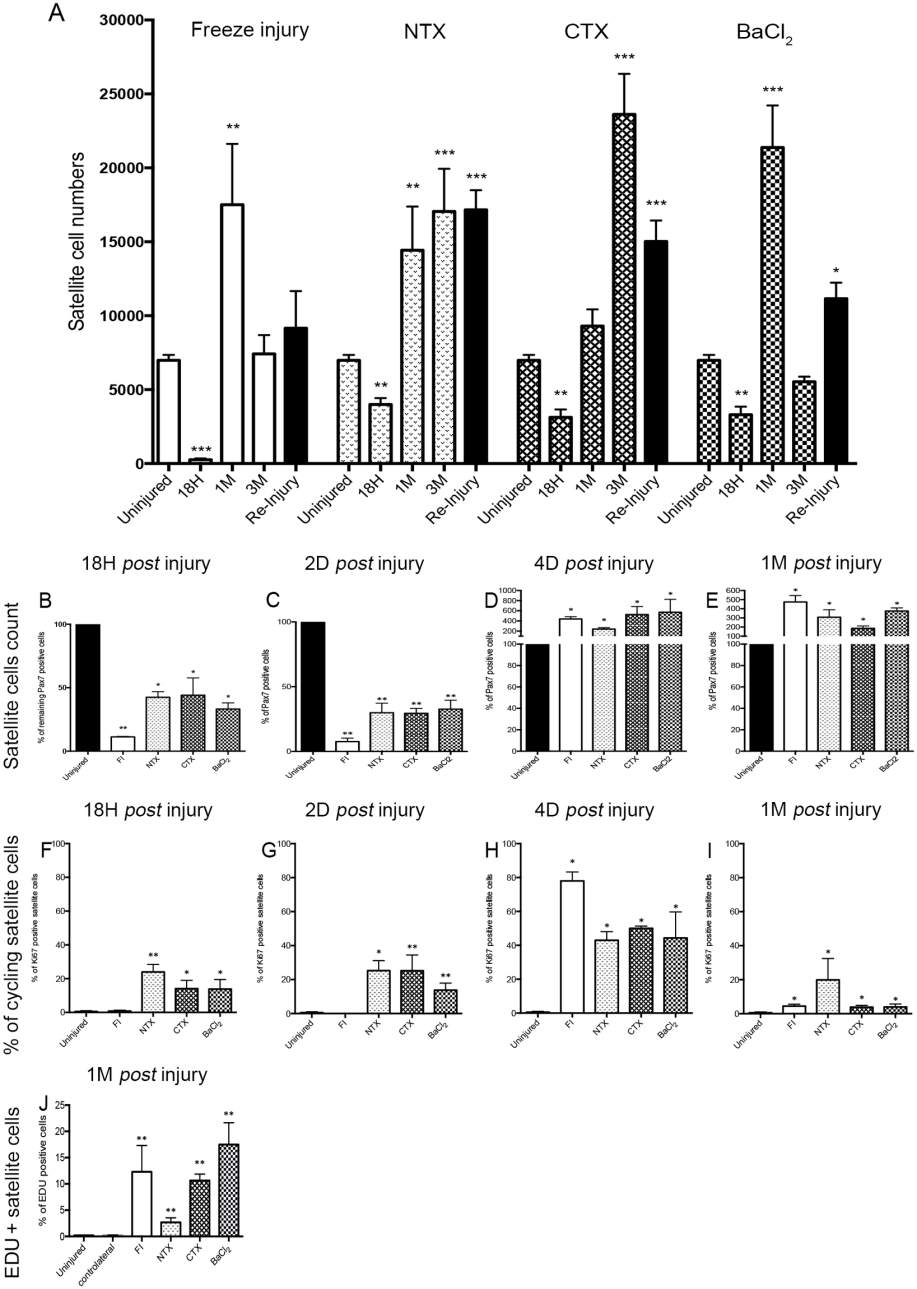
Number of satellite cells and their behaviour in the 4 different injury models. **(A)** Number of satellite cells in uninjured, 18h, 1 month and 3 months post-injury; n = 124 animals (ancillary and new data). The figure also displays the number of satellite cells after re-injury (*i*.*e*. after two successive lesions carried out 28 days apart; displayed in black histograms) n = 5 animals. **(B-E)** Percentage of remaining Pax7 positive cells **(B)** 18h, **(C)** 2 days, **(D)** 4 days and **(E)** 1 month post-injury on TA sections. **(F-I)** Percentage of cycling Ki67 positive satellite cells **(F)** 18h, **(G)** 2 days, **(H)** 4 days and **(I)** 1 month after injury. Data are represented as means±s.d. *p < 0.05; **p < 0.01; ***p < 0.001; no star, statistically non significant.

The four injury models examined here were investigated using a variety of readouts, and the outcome of this analysis is documented in Table 1. The main focus of this study was on the impact of these injury models on the various cell types, and the regeneration process. Given that the *Tibialis anterior* (TA) is the most commonly used muscle for regeneration studies, most of our studies were focused on this muscle. We enumerated satellite cells isolated by FACS from *Tg*:*Pax7nGFP* animals and collected ancillary data from all control experiments for the last year in our lab (Fig 1A). We observed in 6 to 10 week adult C57Bl6/J mice, indistinctly male or female, that one TA contains 7000±400 satellite cells (n = 124 mice) (S2A and S2B Fig). These data were further confirmed on sections (Fig 1B–1E).

A major discriminating feature of a freeze injury (FI), or cryolesion, procedure is the presence of an acute and diffuse necrosis of the muscle tissue. In this model, a “dead zone” is demarcated that lacks viable cells, sparing a zone of viable tissue that will initiate the regeneration process. The extent of the dead zone depends on the number of freezing cycles. Unlike other injury models, freeze-injury allows the investigation of how cells and infiltrate are displaced in a directional manner from the spared tissue into the dead zone in the absence of exogenously injected chemical compounds or toxins. This contrasts with myotoxin- or chemical-induced injuries that have a generalized impact on the entire muscle with a relatively uniform neosynthesis of myofibres and associated structures throughout the tissue. In the case of myotoxins, notexin (NTX) is a phospholipase A2 neurotoxin peptide extracted from *Notechis scutatus* snake venom, and it blocks neuromuscular transmission by inhibition of acetylcholine release. Its hydrolytic activity causes phospholipid hydrolysis of the plasmic membrane, loss of ion gradients, and hypercontraction [15,16]. Cardiotoxin (CTX), a peptide isolated from *Naja nigricollis* venom, is a protein kinase C-specific inhibitor that induces depolarization and contraction of muscle cells and destroys the structure of cell membranes [17]. Both myotoxins provoke an overall destruction of the tissue, but the impact on regeneration is largely dependent on the dose and the batch of myotoxin used. We found that if these used inappropriately, pockets of undamaged myofibres persist thereby increasing the variability of the process of regeneration (data not shown). Finally, BaCl_2_ is the most commonly used method of chemical injury and we found it to have a milder impact on the muscle tissue with patches of uninjured areas depending on the volume used or delivery into the muscle. Here also, the dose and quantity need to be carefully determined to produce homogeneous tissue damage.

### Differential loss of satellite cells in different injury models

#### Freeze-injury

We performed SC counts after different types of injury and noted dramatic differences. For example, following FI (Fig 1A), there was: (i) an early dramatic decrease in SC number at 18h (96% loss by cell sorting and 89% loss evaluating by Pax7 IHC; Fig 1B–1I), and (ii) a two-fold increase, in comparison to control, one month post-FI, in spite of the fact that the muscle tissue appeared to be histologically normal. This difference normalized with time, and 3 months post-FI, SC count was comparable to uninjured muscle. Although satellite cell number increased after a re-injury (two lesions carried out 28 days apart), this did not appear to be statistically significant. These data were further confirmed by histology of sections. This confirmed the initial loss of SC in this model. Interestingly we did not detect any cycling SCs up to 2 days post-injury, but by 4 days post-injury the FI model displayed the highest number of cycling SCs (80%). One month post-injury we observed that 7% of the cells were still cycling. We confirmed this data using the thymidine analogue EdU to evaluate SC proliferation, we noted that 12±5% of SCs were EdU-positive one month after freeze injury compared to 0.2±0.1% in uninjured muscle (Fig 1J). Although the histological organisation of muscle tissue returned to normal a subpopulation of SCs remained in cycle.

#### Notexin

We noted a drop in satellite cell numbers at 18h after NTX injury (Fig 1A), followed by a continuous increase in SC number up to 3 months post-NTX. Two differences were observed in comparison to the FI model: (i) NTX injection was less toxic for satellite cells (the initial drop at 18h post-NTX was less severe than with FI), (4000±1700 SCs; 43% loss by cell sorting and 57% loss evaluating by Pax7 IHC–Fig 1B) and (ii) the satellite cell number continuously increased during muscle regeneration. At the end of the assay period when the muscle was histologically regenerated (3 months post-NTX; also after re-injury), we noted 2–3 times more satellite cells in the regenerated muscles compared to non-injured muscles. Interestingly, the increase in SC number was similar in spite of the fact that FI provoked a greater initial loss of SCs. These data were confirmed on section (Fig 1B–1I).

One month after NTX injury, muscle histology was not returned to the basal state, for example, some calcium deposits persisted. Some SCs were found to be cycling, as determined by the level of EdU incorporation (2.7± 0.8% SC were EdU+ compared to 0.2±0.1% in uninjured muscle. (Fig 1J).

#### Cardiotoxin

Satellite cell numbers significantly decreased during the first 18h after CTX injury (3000±1500 SCs; 55% loss by cell sorting and 56% loss evaluating by Pax7 IHC–Fig 1B), and then increased from this time point. In contrast to FI and NTX injuries, when the muscle tissue was regenerated after 1 month, the number of satellite cells was similar to uninjured muscle, however, SC numbers continued to increase up to 3 months post-CTX, leading to a 3-fold overall increase in satellite cell number compared to the pre-injury state (Fig 1A). After a second round of CTX injury, SC numbers remained elevated by about two fold in comparison to uninjured muscle. These data were confirmed on section (Fig 1B–1I). As with FI and NTX induced injuries, the numbers of cycling SCs remained elevated compared to injured muscle (EdU+, CTX: 10.64±1.2%; Control: 0.2±0.1% uninjured, p = 0.0079, Fig 1J). Importantly, when 50μl of CTX was injected instead of 10μl, we detected a massive loss of SCs 18h post-injury. However 4 days post-injury the number of dividing cells and the behaviour of SCs 1 month post-injury returned to similar levels compared to those with 10μl injections (S6K–S6P Fig).

#### BaCl_2_

As with all of the models above, we observed a loss of SCs at 18h after BaCl_2_ mediated injury (3300±1500 SCs; 53% loss by cell sorting and 67% loss evaluating by Pax7 IHC–Fig 1B), followed by a peak in proliferation to reach 20 000 cells after one month post-BaCl_2_, and then a second drop to less than 6 000 cells 3 months post-BaCl_2_. As with FI, satellite cell numbers also increased after a re-injury (two lesions carried out 28 days apart). The major difference between the two models was in the acute phase following injury where FI appeared to create a less favourable environment for satellite cell expansion. These data were confirmed on section (Fig 1B–1I). Here too, a proportion of SCs remained in cycle once the muscle was regenerated, as attested by the highest level of EdU incorporation in comparison to the other models (BaCl_2_: 17.5±4.1%; Control: 0.2±0.1%, Fig 1J). Importantly, when 50μl of BaCl_2_ was injected instead of 10μl we detected a massive loss of SCs 18h post injury. However, by 4 days post-injury the number of dividing cells and the behaviour of SCs 1 month post-injury was similar to the values observed with 10μl injections (S6K–S6P Fig).

In summary, the presence and disposition of muscle stem cells is radically distinct following each of the injury methods analysed. We have found that FI leads to a 96% loss of SCs 18h post-injury, contrary to other models were there is around 40% loss (Fig 1A). We note that these values can vary between experimenters, volumes used and uniformity of injection into the muscle. Interestingly however, at later time points (3 months post-injury), in the freeze and BaCl_2_ models the number of SCs returned to levels similar to the initial (non-injured) values, whereas in the other toxin (venom) models the number of SCs dramatically increased (Fig 1A). These data were further confirmed on histological sections by Pax7 immunostaining (Fig 1B–1E). When investigating the number of cycling SCs we detected that 20% of cells were dividing (Pax7+/Ki67+) up to 2 days post-injury in the NTX, CTX and BaCl_2_ models (Fig 1F and 1G). This value increased to 50% in those 3 models at 4 days post-injury (Fig 1H). However in the FI model, no cycling cells were detected up to 2 days post-injury but the number of dividing SCs was increased dramatically (80%) by 4 days post-injury. Interestingly, at one month post-injury the number of cycling SC remained elevated compared to non-injured mice (around 5% for FI, CTX and BaCl_2_ and around 20% in NTX model).

### Altered histopathology following muscle trauma

Skeletal muscle regeneration is a highly stereotypical process regardless of the injury method. General features include a peak period where myofibre necrosis occurs, followed by progressive restoration of the tissue by neosynthesized fibres that are centronucleated. In spite of these common features, there were notable differences in satellite cell kinetics remodelling of vasculature, inflammation, fibrosis and the profile of the basal lamina.

#### Freeze-injury

**Fig 2:**
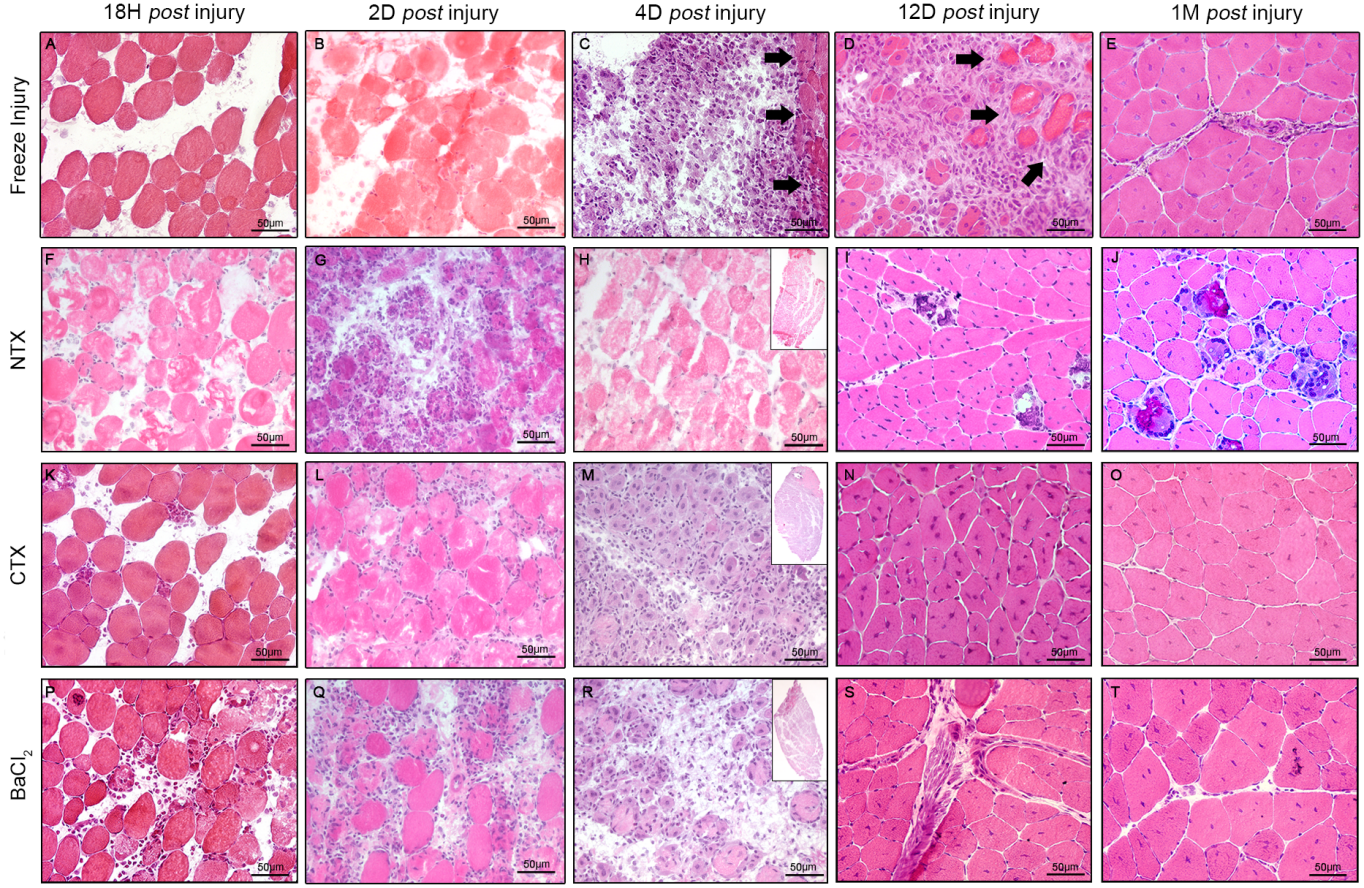
Muscle histology at different time points after injury. Haematoxylin and eosin staining on cryosections. **(A)** 18h, **(B)** 2 days, **(C)** 4 days **(D)** 12 days and **(E)** one month *post* freeze injury. **(F)** 18h, **(G)** 2 days, **(H)** 4 days **(I)** 12 days and **(J)** one month *post* NTX injury. **(K)** 18h, **(L)** 2 days, **(M)** 4 days **(N)** 12 days and **(O)** one month *post* CTX injury. **(P)** 18h, **(Q)** 2 days, **(R)** 4 days **(S)** 12 days and **(T)** one month *post* BaCl_2_ injury. Insets represent whole muscle scan **(HMR)**. Scale bar represents 50 μm.

**Fig 3:**
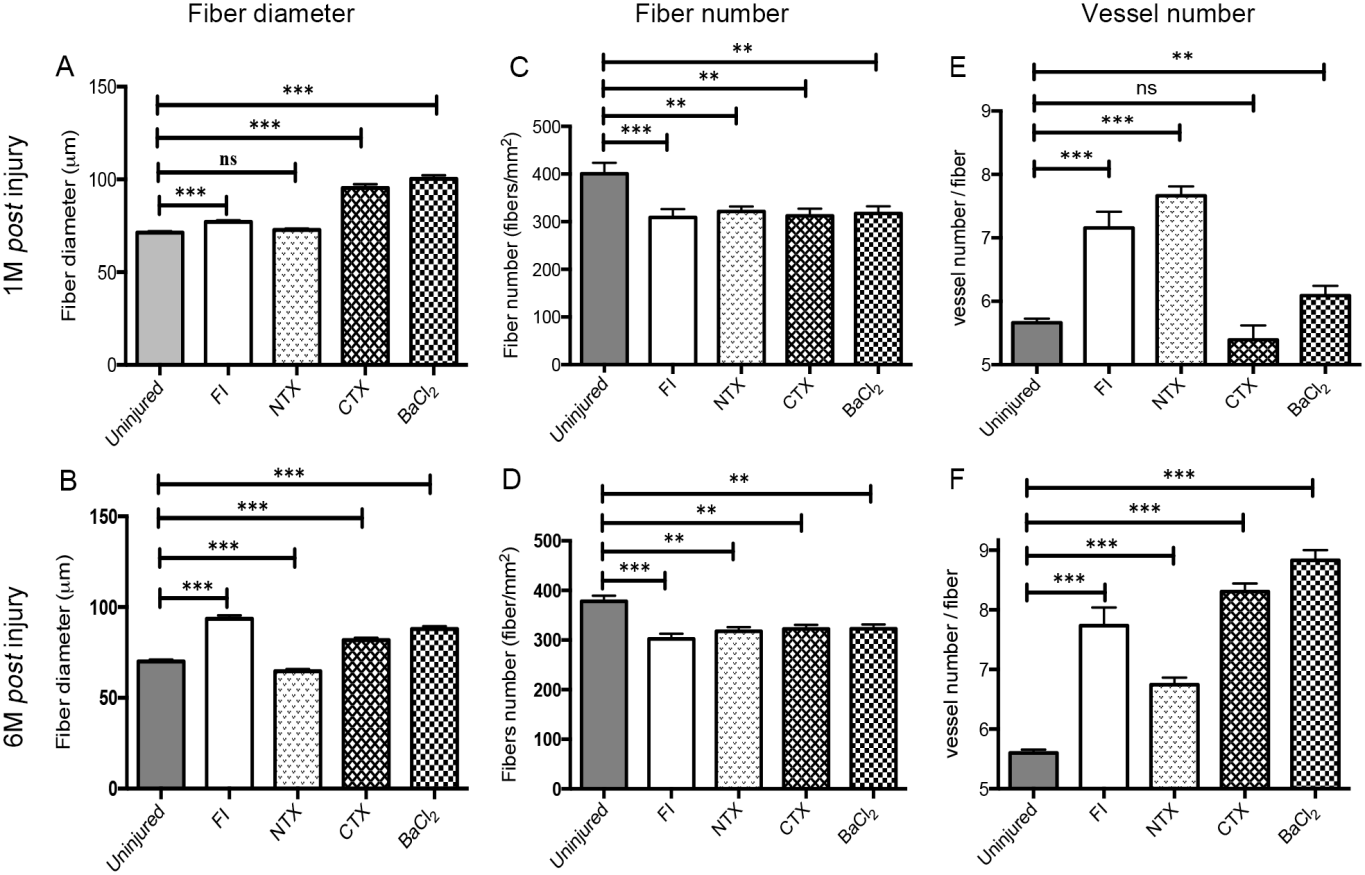
Fibre quantification and vascularization at different time points post injury in the 4 injury models. **(A)** Fibre diameters (expressed in μm) 1 month after injury in all 4 injury models **(B)**, 6 months after injury in all 4 injury models **(C,D)**, 1 and 6 months, respectively, in all injury models. **(E)** Vessel numbers per fibre 1 month after injury in all injury models. **(F)** Vessel numbers per fibre 6 month after injury for all injury models. Data are represented as means±s.d. *p < 0.05; **p < 0.01; ***p < 0.001; ns, statistically non significant.

Eighteen hours post-FI, a diffuse necrosis of myofibres, connective tissue, and blood vessels was observed in the area referred to as the "dead-zone", as distinguished by the absence of viable cells (Fig 2A). Four days post-FI, a regenerative front (Fig 2C, black arrows) appeared at the periphery of the necrotic zone, characterised by infiltration of macrophages and the presence of myoblasts. This regeneration front is a major hallmark of this model. Twelve days post-FI, the regeneration front progressed further, and only a small necrotic zone remained beneath the epimysium (Fig 2D). One month post-FI (Fig 2E), regeneration was complete and myofibres were centrally-nucleated. Interestingly, myofibre size increased (hypertrophy) (77±1 μm *vs*. 71±0.1 μm) whereas fibre number significantly decreased (sarcopenia) 1 month and 6 months post-FI (309±17 vs. 401±22 fibers/mm^2^) when the muscle was expected to have returned to homeostasis (Fig 3A–3D). Interestingly 3 months, 6 months and after a re-injury (two lesions carried out 28 days apart) the muscle appeared to be well regenerated and displayed a normal histology (S3A–S3C Fig)

#### Notexin

Histological analysis was performed on H&E cryosections from 18h to 180d post-notexin injection (post-NTX) (Fig 2F–2J, Table 2).

**Table 2:** Qualitative and semi-quantitative summary of the histological study for the four injury models at the different time points.

	H18				4D				12D				1M				3M				Re-Injury			
	FI	NTX	CTX	BaCL2	FI	NTX	CTX	BaCL2	FI	NTX	CTX	BaCL2	FI	NTX	CTX	BaCL2	FI	NTX	CTX	BaCL2	FI	NTX	CTX	BaCL2
**Necrosis**	++++	++++	++++	+++	+++	+	+	+	-	-	-	-	-	-	-	-	-	-	-	-	-	-	-	-
**Oedema**	+++	+++	+++	+++	+++	++	++	++	+	-	-	-	-	-	-	-	-	-	-	-	-	-	-	-
**Inflamatory infiltration**	+	++	++	+++	++	++++	++++	+++	++	+ GMC	-	-	-	+ GMC	-	-	-	+ GMC	-	-	-	+ GMC	-	-
**Myoblastes**	-	-	-	-	++	+++	+++	+++	++	-	-	-	-	-	-	-	-	-	-	-	-	-	-	-
**Centronucleated fibers**	-	-	-	-	-	-	-	-	+	+++	+++	+++	++++	++++	++++	+++	++++	++++	++++	+++	++++	++++	++++	+++
**Calcium deposits**	-	-	-	-	-	-	-	-	-	+++	-	-	-	++	-	-	-	+	-	-	-	+	-	-
**Vascularisation**	- -	-	-	-	-	+	+	+	+	++	++	++	++	++	++	++	++	++	++	++	/	/	/	/
**Satellite cell numbers (cell sorting in 1 TA)**	259	3995	3129	3303	/	/	/	/	/	/	/	/	17508	14435	9304	21375	7422	17053	23619	5538	9163	17164	15025	11152
**EdU positive cells**	/	/	/	/	/	/	/	/	/	/	/	/	12%	2%	6%	17%	/	/	/	/	/	/	/	/

Results are expressed as a percentage of the tissue infiltrated by inflammatory cells

(- -) virtualy not detected.

(-) rare event.

(+) between 10% and 30%.

(++) between 30% and 50%.

(+++) between 50% and 80%.

(++++) over 80%.

(GMC) presence of syncytial macrophages forming Giant Multinucleated Cells.

(/) not done.

Eighteen hours post-NTX, large areas of myocyte necrosis were observed with no inflammation. The first infiltrating inflammatory cells were detected in association with small myoblasts at 4d post-NTX. At 12 days, muscle appeared to be largely regenerated with centrally-nucleated small basophilic fibres, however multifocal calcium deposits, replacing necrotic myofibres and eliciting foreign body granulomatous reaction at their periphery, were identified. One month post-NTX, these calcium deposits circled by a granulomatous reaction were still present and persisted for 6 months (S3 Fig). Morphometric analysis revealed a sarcopenia 1 month and 6 month post-NTX (NTX: 321±74 fibres; Control: 401±196 fibres, p = 0.0063), a similar myofibre diameter in the two groups 1 month post-NTX (about 72 μm; p = 0.2038) and a slight decrease of myofibre diameter 6-months post-NTX (Fig 3A–3D).

#### Cardiotoxin

Histological analysis was performed on H&E cryosections from 18h to 180d post-cardiotoxin injection (post-CTX) (Fig 2K–2O, Table 2). Histological lesions were similar to those observed in NTX-injury, with marked and extensive necrosis 18h post-CTX, infiltration of inflammatory cells from day 4, and complete regeneration 1-month post-CTX. The major difference was the absence of mineralisation nor granulomatous inflammatory reaction in the muscle tissue after CTX injection. Morphometric analysis revealed a sarcopenia 1-month and 6-month post-CTX (CTX: 312±89 fibres; Control: 401±196 fibres; p = 0.0063), and an hypertrophy compared to control animals, in contrast to what was observed with the NTX model (CTX: 95±2 μm; Control: 71±22 μm; p<0.0001) (Fig 3A–3D). Interestingly 3 months, 6 months and after a re-injury the muscle appeared to be well regenerated and displayed a normal histology (S3G–S3I Fig).

#### BaCl_2_

Histological analysis was performed on H&E cryosections from 18h to 180d post-BaCl_2_ injection (Fig 2P–2T, Table 2). Histological lesions were similar to those observed following CTX-injury, with marked and extensive necrosis at 18h, and complete regeneration 1-month post-BaCl_2_, without mineralisation nor granulomatous inflammatory reaction. After 1 and 6 months of regeneration, as with the other models, morphometric analysis revealed a sarcopenia (BaCl_2_: 317±104 fibres; Control: 401±196 fibres, p = 0.0071) and an hypertrophy compared to control animals (BaCl_2_: 100±2 μm; Control: 71±22 μm, p<0.0001) (Fig 3A–3D). Interestingly 3 months, 6 months and after a re-injury the muscle seems to be well regenerated and displays a normal histology (S3J–S3L Fig).

### Inflammation

**Fig 4:**
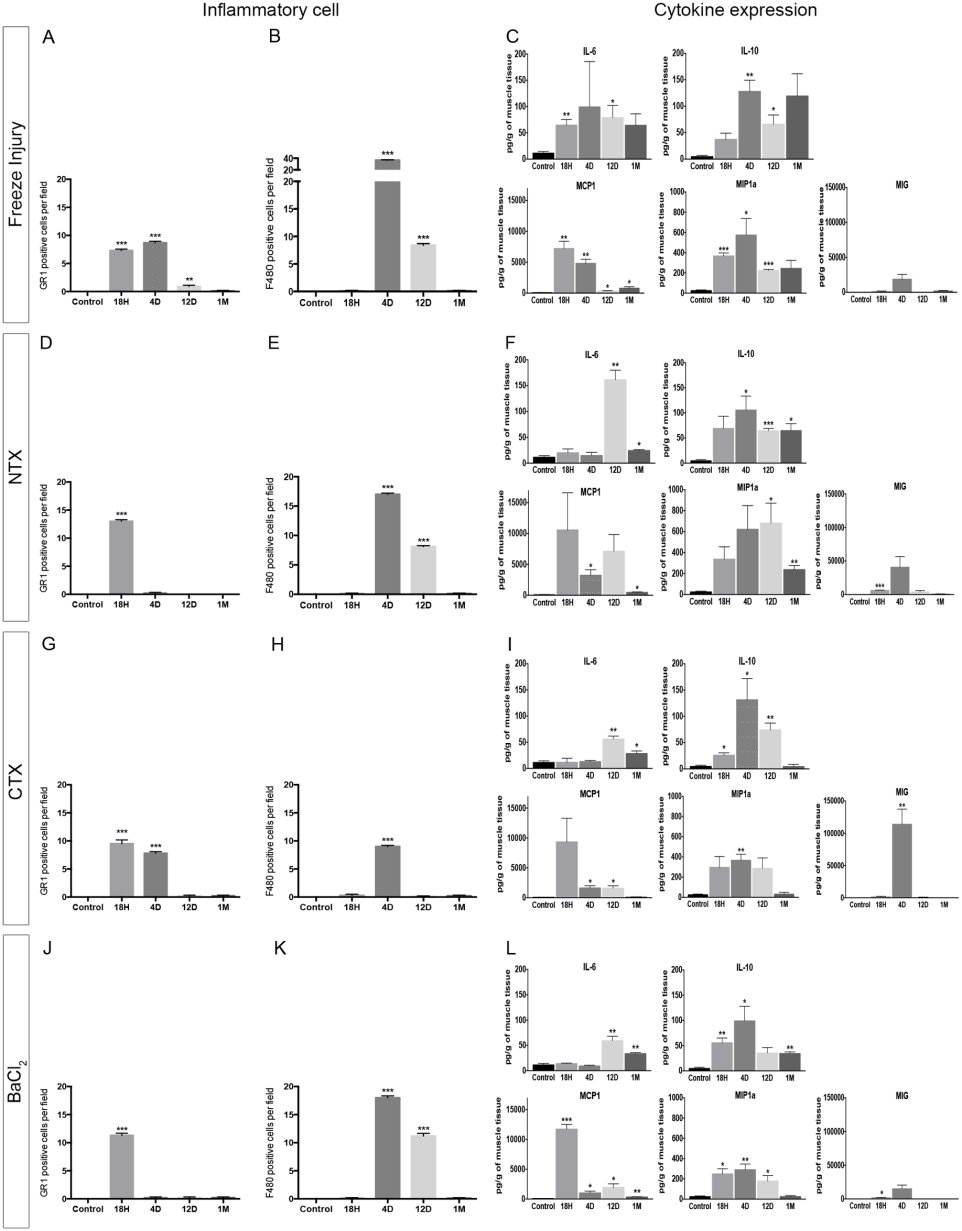
Characterization of inflammation after injury in the 4 injury models. **(A,D,G,J)** Neutrophil (GR1+ cells) quantifications (expressed as number of cells per ten microscopic fields at 40X) in the FI (**A**), NTX (**D**), CTX (**G**) and BaCl_2_ (**J**). **(B,E,H,K)** Macrophage (F4/80+ cells) quantifications (expressed as number of cells per field) in the FI (**B**), NTX (**E**), CTX (**H**) and BaCl_2_ (**K**). Data are represented as means±s.d. *p < 0.05; **p < 0.01; ***p < 0.001; no star, statistically non significant. (**C,F,I,L**) Luminex (multiplex assay) measuring the levels of cytokines in pg/g of muscle tissue, 18h, 4 days, 12 days and 1 month post-injury in the freeze injury model (**C**), the NTX (**F**), the CTX (**I**), and the BaCl_2_ (**L**). Selected cytokines (IL6; IL10; MCP1; MIP1a and MIG) are displayed for each injury model. Data are represented as means±s.d.

We carried out a global analysis of inflammatory processes during muscle regeneration by investigating inflammatory cell infiltrates in parallel to cytokine and chemokine expression (Fig 4 and Table 3).

**Table 3:** Summary of the inflammatory infiltrate in the different injury models (cell number per 10 microscopic fields).

	H18				4D				12D				1M				3M				Re-Injury			
	FI	NTX	CTX	BaCL2	FI	NTX	CTX	BaCL2	FI	NTX	CTX	BaCL2	FI	NTX	CTX	BaCL2	FI	NTX	CTX	BaCL2	FI	NTX	CTX	BaCL2
**GR1**	7	10	13	12	9	8	0	0	1	0	0	0	0	0	0	0	0	0	0	0	0	0	0	0
**B220**	0	0	0	0	0	0	0	0	0	0	0	0	0	0	0	0	0	0	0	0	0	0	0	0
**CD3**	0	0	0	0	0	0	0	0	0	0	0	0	0	0	0	0	0	0	0	0	0	0	0	0
**F4/80**	0	0	0	0	36	17	9	18	8	8	0	11	0	9	0	0	0	10	0	0	5	13	0	0

The number in the Table indicate the numbers of cells per section enumerated in the 4 different injury models.

#### Freeze-injury

Almost no T (CD3+) or B (B220+) cells were detected in the muscle, during the entire regeneration process. However, as soon as 18h post-FI in the extensive necrotic tissue, only neutrophils (Gr1+) were identified (Fig 4A), most often at the periphery of necrotic fibres. Four days post-FI, neutrophils were still detectable, but they were no longer present at later time points. Macrophages (F4/80+) appeared at 4d post-FI, however, they later decreased in number, and at 1-month post-FI (Fig 4B), almost no inflammatory cells were observed in the regenerated tissue.

Cytokine/chemokine quantitation during injury showed that the level of both pro-inflammatory and anti-inflammatory cytokines increased from the early stages of regeneration process to the late time points, 1 month post-FI, without returning back to expression levels observed in control mice (Fig 4C). An early increase of IL-6 from 18h (FI: 65±19 pg/g; Control: 11±5 pg/g, p = 0.0087) was observed post-FI contrary to other injury models. Similarly, the peak of monocyte chemoattractant protein 1 (MCP-1) occurs early during the survey (18h) while those of Macrophage Inflammatory Proteins α (MIP1α) and Monokine induced by gamma interferon (MIG) occurred on day 4.

#### Notexin

As with freeze injury, almost no T (CD3+) or B (B220+) lymphocytes were detected in the muscle during the whole regeneration process, the most prominent cells being neutrophils (at the early time points) and macrophages (from day 4 post-NTX) (Fig 4D). The major detectable difference between FI and NTX models of lesion was the kinetics of cell infiltration. The peak of neutrophil infiltration was at 18h post-NTX (in contrast to day 4 for FI), and a significant number of macrophages could still be detected in the muscle tissue at day 12 and 1 month post-NTX (Fig 4E), when almost no inflammation could be detected after freeze injury at the same time points.

Cytokine/chemokine quantitation during injury showed that expression of both pro-inflammatory and anti-inflammatory cytokines increased at the early stages of regeneration process but then returned back to control levels except for IL-10 and MIP1α where levels continued to remain elevated (Fig 4F). An important peak of IL-6 (NTX: 161±32 pg/g; Control: 11±5 pg/g, p = 0.013) was observed 12 days post-NTX, which decreased 1-month post-injury (NTX: 24±3 pg/g; Control: 11±5 pg/g, p = 0.0233).

#### Cardiotoxin

Kinetics of inflammatory cell recruitment was similar in all of the injury models we studied, but inflammatory lesions were more restricted in time in the CTX model compared to the others. Neutrophils were observed only 18h post-CTX (Fig 4G), and macrophages were detected only at day 4 (Fig 4H). No other inflammatory cell was identified at the other time points, and T and B lymphocytes did not appear to have a major impact on muscle regeneration as they were not detected in the tissue at any time point (Table 3). Expression of both pro-inflammatory and anti-inflammatory cytokines increased weakly at the early stages of the regeneration process except for MCP1, but they then returned back to control levels for all cytokines except for IL-6 that remained high 1 month post-CTX (Fig 4I). The most important peak of MIG as compared to any other models (CTX: 113839±40760 pg/g; Control: 0 pg/g; p = 0.0084) was observed 4 days post-CTX, and this returned to normal levels 12 days post-injury.

#### BaCl_2_

BaCl_2_ injury displayed similarities with the other models: (i) almost no lymphocytes were detected during regeneration, (ii) neutrophils and macrophages were the most prominent inflammatory cells in early and late stages, respectively, (iii) neutrophil infiltration was largely restricted to the very acute phase (Fig 4J), and (iv) the kinetics of macrophage infiltration was similar to the FI model (peak of infiltration at day 4 and decreased infiltration at day 12) (Fig 4K). In contrast to FI, we noted more infiltrating macrophages (11.4 *vs* 8.6 cells/field) in the muscle tissue at day 12, in the BaCl_2_ model. Expression of both pro-inflammatory and anti-inflammatory cytokines increased at the early stages of regeneration, particularly with a peak of MCP-1 observed at 18h, and then returned back to control expression levels 1 month post-BaCl_2_ except for IL6, IL-10 and MCP1 where levels remained elevated (Fig 4L). In this model, the response to IL-10 was modest and with a peak observed 4 days after the injury as with the other models (BA: 99±29pg/g; Control: 4±2 pg/g, p = 0.0316).

### Remodelling of vasculature

#### Freeze-injury

To examine the extent of damage to vasculature, we investigated the capillary section number per myofibre (called myofibre “capillarisation”) using Laminin/CD31 immunolabeling (S4A–S4D Fig). One-month post-FI, we observed an increase in fibre capillarisation (FI: 7.2±0.3 capillary sections/fibre; control: 5.7±1.9 capillary sections/fibre), which was maintained up to six months post-FI (7.7±0.3) (Fig 3E and 3F).

**Fig 5:**
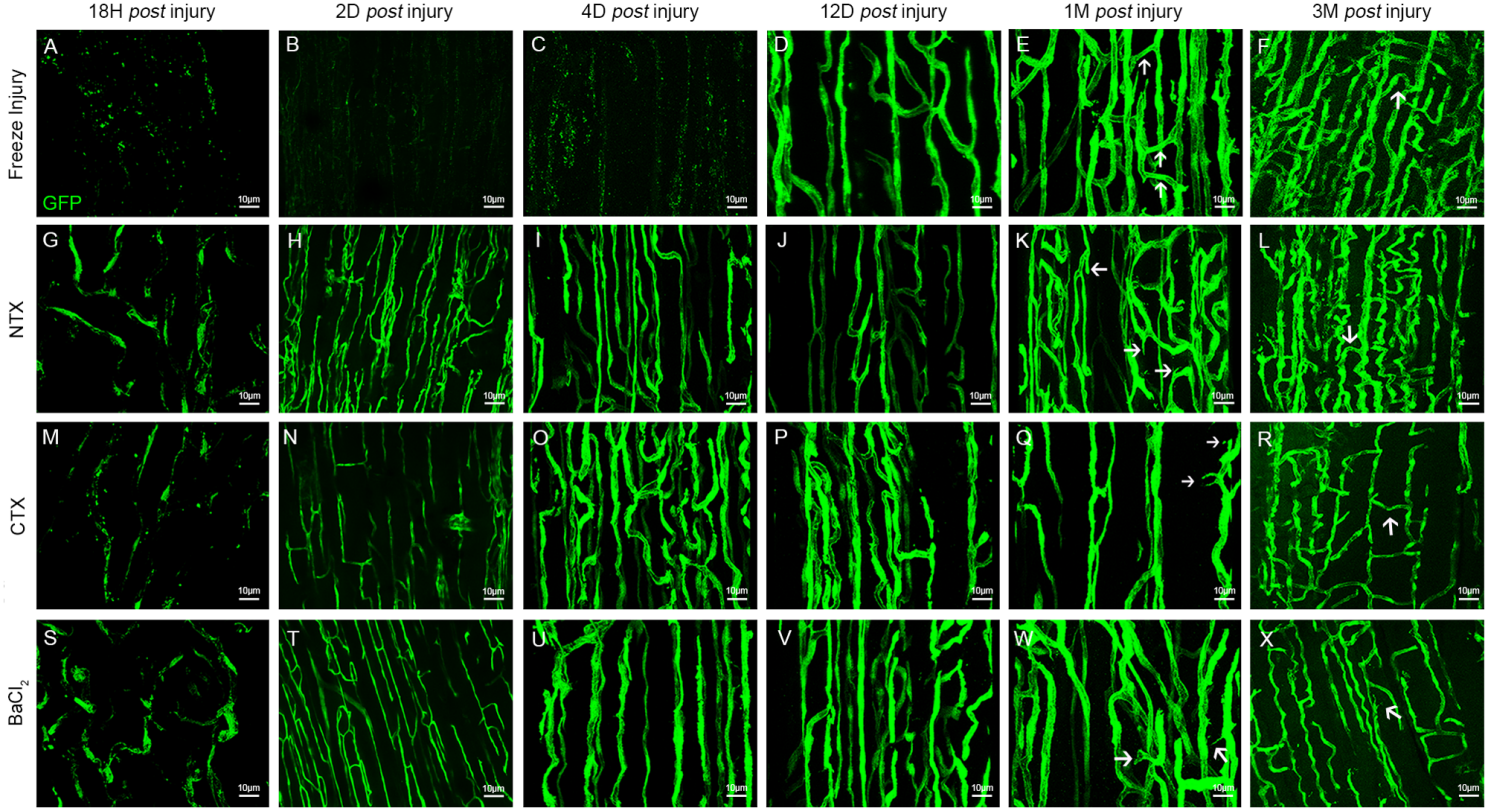
Three dimensional analysis of vessels at different time points in all 4 injury models. Images show blood vessel organisation in 3D after z-stack reconstitutions of scanned sectioned TA from *Flk1*^*GFP/+*^ mouse. **(A-F)** Vessel organisation in the freeze injury, 18h (**A**), 2 days (**B**), 4 days (**C**), 12 days (**D**), 1 month (**E**) and 3 months (**F**) post injury. **(G-L)** Vessel organisation in the NTX injury, 18h (**G**), 2 days (**H**), 4 days (**I**), 12 days (**J**), 1 month (**K**) and 3 months (**L**) post injury. **(M-R)** Vessel organisation in the CTX injury, 18h (**M**), 2 days (**N**), 4 days (**O**), 12 days (**P**), 1 month (**Q**) and 3 months (**R**) post injury. **(S-X)** Vessel organisation in the BaCl_2_ injury, 18h (**S**), 2 days (**T**), 4 days (**U**), 12 days (**V**), 1 month (**W**) and 3 months (**X**) post-injury. Arrows pointing anastomoses. Scale bars represents 10 μm.

To assess the extent of the vascular network and its organisation, we employed 3D-imaging using *Flk1*^*GFP/+*^ mice that display green fluorescent endothelial cells (Fig 5A–5F). Confocal microscopy analysis revealed, (i) a fluorescence altered 18h post-FI with a split vessel network that appeared “ghost-like”. Some areas were completely devoid of endothelial cells, (ii) formation of new blood vessels in the regeneration front 4d post-FI with a disorganized vascular network, (iii) formation of a well organized new vascular network, but less dense than in the control, and (iv) a complete restoration of the vascular network 1 month post-FI, with an increase in capillary density including numerous anastomosis inappropriately tortuous, persisting 3 months after injury (Fig 5F).

#### Notexin

Capillary density and morphology were evaluated both in 2D (CD31/Laminin immunofluorescence) and 3D (using *Flk1*^*GFP/+*^ mice). As with freeze injury, the microvascular network exhibited alterations 18h post-NTX, but this was less intense than in the FI model. Four and twelve days after injury, the vascular network was partially restored with a notable disparity in density. Vessels had a smaller diameter than in control, and no anastomosis was observed (Fig 5I and 5J). The vascular network returned to normal 1 month post-NTX (Fig 5K), with a detectable increase in overall capillarisation persisting for 6 months (NTX: 7.7±0.1 capillaries/myofibre; Control: 5.7±1.9 capillaries/myofibre), and an increase in micro-anastomoses, persisting 3 months after injury (Figs 5L, 3E and 3F).

#### Cardiotoxin

Analysis of capillary density and morphology revealed an initial destruction of the capillary network followed by angiogenesis and complete regeneration 1 month after injury (S4I–S4L Fig), as with the other models. The different morphometric parameters evaluated to study the microvascular network organisation revealed similar alterations in vessels compared to the NTX model. Four days after injury, the vascular network was dense and more tortuous than in the control. Twelve days after injury, the vascular network was well organized, however grouping of vessels was noted. There was vessel remodelling 1 month after injury where the vascular network appeared less dense than in the control (Fig 5M–5R). Moreover, contrary to other models, fibre “capillarisation” was unchanged 1 month after injury (Fig 3E and 3F).

#### BaCl_2_

As observed with NTX, the microvascular network exhibited alterations 18h post-BaCl_2_, but that was less intense than in the FI model. Four and twelve days after injury, the vascular network was partially restored with a disparity in density. Vessels had smaller diameters than control and no anastomosis was detected (Fig 5U and 5V). The vascular network returned to normal 1-month *post*-BaCl_2_ (Fig 5W). Blood capillaries were altered at 18h, neoangiogenesis was detected from day 4, and the microvascular network appeared to be fully regenerated 1 month post-injury, with an increase of anastomosis persisting 3 months after injury (Fig 5X). As with FI and NTX injuries, myofibre capillarisation increased 1 month post-BaCl_2_ (BaCl_2_: 6.1±0.1 capillary per myofibre; Control: 5.7 ±1.9 capillary per myofibre; p<0.0046; Fig 3E), and it persisted at 6 months after injury (Fig 3F).

### Fibrosis

**Fig 6:**
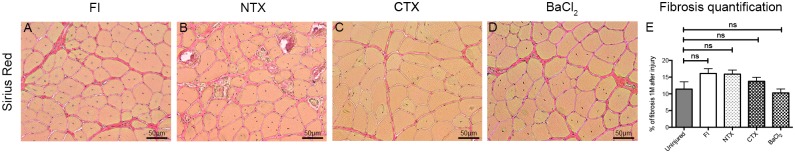
Characterization of fibrosis after injury. **(A-D)** Sirius Red staining (collagen deposits)1 month after injury in all 4 injury models. **(E)** Percentage of fibrosis per section 1 month after injury compared with non-injured control. No statistically significant differences detected among the 4 models. ns; non significant.

Fibrosis, characterised by migration and proliferation of fibroblasts into the site of injury and excessive production of extra-cellular-matrix (ECM) proteins by these cells replacing normal tissue structure, is the hallmark of compromised or failed muscle regeneration. For all injury models, no appreciable fibrosis was observed 1-month post-injury except for the freeze injury model where some endomysial collagen deposition was detected 1-month post-FI (control: 11±2% of total muscle area; FI: 16±1%, 1 month after FI; Fig 6A–6E).

### Basal lamina

Using immunofluorescence, we showed that although the expression and organisation of laminin 1, a major component of muscle basal lamina, was altered particularly in the FI model, a ghost of laminin 1 was consistently preserved around each muscle fibre 18h post-injury. This lamina was reconstituted in all models by 1 month post-injury (S4A–S4P Fig).

## Discussion

Skeletal muscle, skin and liver have been used as reference tissues for the study of tissue regeneration. In the muscle field, this interest translated in an intense research activity on muscle stem cells and their properties [1,18–21]. Although more information is becoming available on the muscle stem cell population, studies on other tissue constituents are largely lacking. In 2014, over 9,200 publications referring to muscle injuries were referenced in Pubmed (NCBI). Almost half of these articles included *in vivo* studies and experimentally induced muscle injury. Among these, the most often used injury models were: CTX (75%), NTX (11%), BaCl_2_ (10%) and freeze-injury (4%).

Different experimental paradigms and injury models have been used seemingly arbitrarily in the literature rendering some comparisons between laboratories problematic. Therefore, we focused our comparative analysis on the major parameters that are examined during muscle regeneration with the aim of establishing benchmarks and readouts that can be used in future studies. Generally, we found that the initial phase following injury is critical, influencing the overall outcome of the regeneration. Our analysis also showed that the regeneration process restored the tissue in all models investigated. However, the pathophysiologic mechanisms involved varied significantly depending on the model, and we identify major differences in the kinetics of regeneration, involvement of vascular network, and satellite cell behaviour.

For example, intramuscular injections of CTX, NTX and BaCl_2_ systematically provoked a monophasic necrosis of the muscle tissue, followed immediately by sequential and synchronous regeneration characterised by neutrophil and macrophage infiltration. Myoblasts then appeared and they fused to form myofibres. In contrast, the freeze-injury model was characterised by asynchronous regeneration with the presence of a regeneration front, in which all the different regeneration steps were present simultaneously as cells penetrated into the dead zone. As far as inflammation was concerned, although the kinetics appeared to be identical for all models, notable differences were observed in the production of cytokines: in the CTX group, cytokine expression returned to normal as soon as the muscle was histologically regenerated. In contrast, in the other models, expression of cytokines was never restored to normal levels thereby suggesting persistent inflammation in spite of a normal histological appearance of the muscle tissue.

Satellite (stem) cells play a crucial role in muscle regeneration [22,23], however it has been clearly demonstrated that involvement of the satellite cell macro- and micro-environment (niche) [24] and interactions with fibroblasts [7] and inflammatory cells (mostly macrophages) are paramount for efficient regeneration [25–29]. In our study, we report different alterations in the satellite cell microenvironment depending on the model. These include severe vascular lesions especially in FI and granulomatous inflammatory reaction after NTX injection. These findings explain to some extent the differences in regeneration kinetics that we observed. Other critical parameters include satellite cell survival and expansion, cytokine types and levels of expression, and inflammation kinetics. Clearly these parameters should be taken into account for any study, and we propose that they will also impact the behaviour of satellite cells or other cell types following transplantations.

Regarding satellite cell behaviour following injury, we demonstrated that all of the injury models tested resulted in a major loss of satellite cells, and this varied significantly in severity. Freeze injury being the only non-chemical method would suffer to a lesser extent compared to NTX and CTX where batch to batch differences can provoke variable losses in satellite cells (present study and [2]). In addition, we showed that the injection of different volumes of toxins can have an impact on the area of the injured muscle and the behaviour of satellite cells. Indeed higher volumes of toxins injected lead to higher SC death and a delay in SC division. We also found that the triple induced freeze method used here can provoke different levels of destruction depending on the force and extent of contact with the liquid nitrogen rod. In the present study, about 95% of satellite cells were lost, in contrast to NTX, CTX and BaCl_2_ where almost 60% satellite cells survived, when 10μl of toxin were injected. For the chemical-induced and freeze models, they can be scaled down to a focal injury (data not shown), however, the chemical methods would have a diffusion gradient of the product as opposed to the freeze injury. Thus, the choice of a specific model impacts on satellite cell physiology and self-renewal potential. Indeed, 1 month post-injury, a higher number of satellite cells was detected in all models. The underlying cause of this increase requires further investigation, however the cells are functional, as serial rounds of grafting and injury yielded efficient muscle regeneration for each regeneration cycle [30–34]. In these studies the environment plays a key role in the behaviour of the satellite cells and the model of injury will greatly impact the environment, thus consistently choosing the same model and knowing the environment in which the cells will be grafted is key to interpret and compare the results.

It is generally thought that by 3–4 weeks following muscle injury, regeneration restores the muscle to homeostasis. Surprisingly however, although muscle organisation does appear histologically normal by 28 days post-injury, we noted that satellite cells continued to cycle at different rates in the different models examined. Several laboratories have reported heterogeneities in the satellite cell population [34–39]. Indeed, those expressing the paired homeobox transcription factor Pax7 at low levels (Pax7^low^) during quiescence are more poised for activation, whereas Pax7 high expressing cells (Pax7^high^) appear to be more stem-like and are in a deeper state of quiescence called dormancy [34]. It would be interesting to determine which subpopulation is activated following different forms of injury, and when dormant satellite cells acquire this property after homeostasis.

Remodelling of the vascular network has been largely ignored in the field of muscle regeneration, yet this clearly plays an important role in skeletal muscle regeneration as, it impacts on the distribution of recruited inflammatory cells and regeneration-related factors (growth factors, cytokines, chemokines), and as the paracrine effect between satellite and endothelial cells affects the regenerative process [40,41]. Using *Flk1*^*GFP/+*^ mice, we demonstrated that all injury models resulted in destruction of the vasculature, especially FI. This severe lesion, in contrast to what was observed in the other models, resulted in the total destruction of the vascular network, leading to a delay and an incomplete regeneration even one month post-injury.

It has been demonstrated that about 85% of satellite cells are located close to blood capillaries [40]; this specific location could have an impact on satellite cell subpopulations where variations in oxygen concentration can occur [14,42]. Satellite cells located in hypoxic niches are indeed less differentiated [43] and it was previously shown *in vitro* that hypoxia (<3% of oxygen) promotes satellite cell proliferation and differentiation [44]. Thus vasculature remodelling should be taken into account following transplantations where access to the blood milieu could impact on their phenotype and behaviour.

Notably, in the notexin-treated group, as soon as 12 days post-injury, multifocal calcification of necrotic myofibres appeared eliciting a peripheral granulomatous reaction with multinucleated giant cells. These mineralised fibres remained even 6 months post-injury, as the chronic recruitment and activation of macrophages, even when the spared muscle tissue is completely regenerated. This multifocal chronic inflammation is likely to be responsible for alterations of the cytokine/chemokine expression profile in the regenerated muscle, in comparison to the other injury models. The behaviour of resident or transplanted satellite cells would, for example, be influenced by the higher level of IL6 which would favour differentiation over self-renewal [45–47].

## Conclusion

The muscle injury models examined here are extensively used in the literature to study tissue regeneration and stem cell properties, however our studies show that the nature of the model of injury should be chosen carefully depending on the experimental design and desired outcome. Although in all models the muscle regenerates completely, the trajectories of the regenerative process vary considerably. Furthermore, we show that histological parameters are not wholly sufficient to declare that regeneration is complete as molecular alterations (*e*.*g*. cytokine levels) could have a major impact on muscle stem and stromal cell behaviour.

## Supporting Information

S1 Fig Muscle freeze injury procedure. **Freeze Injury**: After skin incision (**A**) and muscle exposition (**B**) the *Tibialis* anterior was frozen with three consecutive cycles of freeze-thawing by applying for 15 sec a liquid nitrogen cooled metallic rod (**C, D**). The skin was then sutured (**E**).
(TIF)

S2 Fig Characterization of normal muscle satellite cells, histology, basal lamina and vessels. **(A)** Satellite cell counts by flow cytometery (*Tg*:*Pax7nGFP* mouse). GFP+ cells are the satellite cells; PI is propidium iodide to exclude dead cells. **(B)** Number of satellite cells in control *Tibialis anterior* muscle of n = 124 animals (ancillary and new data) by flow cytometry. **(C, D)** Haematoxylin and eosin stain of control, non-injured muscle. Scale bar represent 50 μm. **(E)** Control, uninjured TA muscle displaying vessel (CD31, red) and laminin (green) immunolabeling. Scale bar represent 50 μm. **(F)** Control uninjured TA muscle 3D imaging of microvascular network (*Flk1*^*GFP/+*^ mouse). Scale bar represent 10 μm.
(TIF)

S3 Fig Histological study 3 and 6 months after injury and reinjury. Haematoxylin and eosin staining on cryosections. **(A)** 3 months, **(B)** 6 months and **(C)** one month after freeze reinjury. **(D)** 3 months, **(E)** 6 months and **(F)** one month after NTX reinjury. **(G)** 3 months, **(H)** 6 months and **(I)** one month after CTX reinjury. **(J)** 3 months, **(K)** 6 months and **(L)** one month after BaCl_2_ reinjury. Scale bar represents 50 μm.
(TIF)

S4 Fig Characterization of vessels and basal lamina after injury in the 4 injury models. All images show blood vessel organisation in 2D (CD31 red / laminin green immunohistochemistry). **(A)** 18h, **(B)** 4 days, **(C)** 12 days and **(D)** one month *post* freeze injury. **(E)** 18h, **(F)** 4 days, **(G)** 12 days and **(H)** one month *post* NTX injury. **(I)** 18h, **(J)** 4 days, **(K)** 12 days and **(L)** one month *post* CTX injury. **(M)** 18h, **(N)** 4 days, **(O)** 12 days and **(P)** one month *post* BaCl_2_ injury. Scale bar represent 50 μm.
(TIF)

S5 Fig Aperture of the skin (sham). (**A-C**) Haematoxylin and eosin stain control (**A**), 18h (**B**), and 1 month (**C**), after aperture of the skin. **(D-F)** Sirius Red staining (collagen deposits) on control (**D**), 18h (**E**), and 1 month (**F**), *post* open skin. (**G-I**) Immunohistochemistry of CD31 (red) and Laminin (green) in the freeze injury model, control (**G**) 18h (**H**) and 1 month (**I**) after aperture of the skin. Scale bar represent 50 μm.
(TIF)

S6 Fig Injury models 50 μL injection. Haematoxylin and eosin staining on cryosections. **(A)** 18h, **(B)** 4 days and **(C)** one month after CTX injury. **(D)** 18h, **(E)** 4 days and **(F)** one month after BaCl_2_ injury. Scale bar represents 50 μm. Images show blood vessel organisation in 3D after z-stack reconstitutions of scanned sectioned TA from *Flk1*^*GFP/+*^ mouse. Vessel organisation after freeze injury 18h (**H**) and BaCl_2_ injury 18h (**I**). Scale bar represents 10 μm. **(J)** Vessel numbers per fibre 1 month after injury 50μL in all injury models. **(K-M)** Percentage of remaining Pax7 positive cells **(K)** 18h, **(L)** 4 days and **(M)** 1 month post-injury on TA sections. **(N-P)** Percentage of activated Ki67 positive satellite cells **(N)** 18h, **(O)** 4 days and **(P)** 1 month after injury. Data are represented as means±s.d. *p < 0.05; **p < 0.01; ***p < 0.001; no star, statistically non significant.
(TIF)

S7 Fig Summary of the freeze injury model regeneration process. (**A-D**) Haematoxylin and eosin stain 18h (**A**); 4 days, arrows indicate regeneration front (**B**); 12 days arrows indicate regeneration front (**C**) and 1 month (**D**) post-injury. Scale bar represent 50 μm (**E-H**) Immunohistochemistry of CD31 (red) and Laminin (green) in the freeze injury model, 18h (**E**), 4 days (**F**), 12 days (**G**) and 1-month (**H**) post-injury. Scale bar represent 50 μm (**I-L**) Images show blood vessel organisation in 3D 18h (**I**), 4 days (**J**), 12 days (**K**), 1-month (**L**) post-injury. Scale bar represent 10 μm, arrows indicate anastomoses. (**M-P**) Count of the number of inflammatory cells per section 18h, 4 days, 12 days and 1 month post-injury. (**M**), number of CD3+ cells; (**N**) number of B220+ cells; (**O**) number of Gr1+ cells; (**P**) number of F4/80+ cells. ***P < 0.001; no star, statistically non significant. (**Q**) Luminex (multiplex assay) measuring the levels of cytokines in pg/g in control, 18h, 4 days, 12 days and 1 month post injury. Selected cytokines are displayed (IL6 blue, IL10 green, IL12p40 yellow, IL12p70 red, MCP1 grey, MIP1a orange, MIP1b black. (**R**) number of satellite cells, counted by cytometry in one specific TA muscle in the control (non-injured), 18h, 1 month, 3 month and 28 days after re-injury. **p < 0.01; ***p < 0.001; no star, statistically non significant.
(TIF)

S8 Fig Summary of NTX injury model regeneration process. (**A-D**) Haematoxylin and eosin stain 18h (**A**); 4 days, (**B**); 12 days (**C**) and 1 month (**D**) post-injury. Scale bar represent 50 μm (**E-H**) Immunohistochemistry of CD31 (red) and Laminin (green) in the NTX injury model, 18h (**E**), 4 days (**F**), 12 days (**G**) and 1-month (**H**) post injury. Scale bar represent 50 μm (**I-L**) Images show blood vessel organisation in 3D 18h (**I**), 4 days (**J**), 12 days (**K**), 1-month (**L**) post-injury arrows indicate anastomoses. Scale bar represent 10 μm. (**M-P**) Count of the number of inflammatory cells per section 18h, 4 days, 12 days and 1 month post-injury. (**M**), number of CD3+ cells; (**N**) number of B220+ cells; (**O**) number of Gr1+ cells; (**P**) number of F4/80+ cells. ***p < 0.001; no star, statistically non significant. (**Q**) Luminex (multiplex assay) measuring the levels of cytokines in pg/g in control, 18h, 4 days, 12 days and 1 month post-injury. Selected cytokines are displayed (IL6 blue, IL10 green, IL12p40 yellow, IL12p70 red, MCP1 grey, MIP1a orange, MIP1b black. (**R**) number of satellite cells, counted by cytometry in one specific tibialis anterior muscle in the control (non-injured), 18h, 1 month, 3 month and 28 days after re-injury. **p < 0.01; ***p < 0.001; no star, statistically non significant.
(TIF)

S9 Fig Summary of CTX injury model regeneration process. (**A-D**) Haematoxylin and eosin stain 18h (**A**); 4 days, (**B**); 12 days (**C**) and 1 month (**D**) post-injury. Scale bar represent 50 μm (**E-H**) Immunohistochemistry of CD31 (red) and Laminin (green) in the CTX injury paradigm, 18h (**E**), 4 days (**F**), 12 days (**G**) and 1-month (**H**) post-injury. Scale bar represent 50 μm (**I-L**) Images show blood vessel organisation in 3D 18h (**I**), 4 days (**J**), 12 days (**K**), 1-month (**L**) post-injury; arrows indicate anastomoses. Scale bar represent 10 μm. (**M-P**) Count of the number of inflammatory cells per section 18h, 4 days, 12 days and 1 month post-injury. (**M**), number of CD3+ cells; (**N**) number of B220+ cells; (**O**) number of Gr1+ cells; (**P**) number of F4/80+ cells. ***p < 0.001; no star, statistically non significant. (**Q**) Luminex (multiplex assay) measuring the levels of cytokines in pg/g in control, 18h, 4 days, 12 days and 1 month post injury. Selected cytokines are displayed (IL6 blue, IL10 green, IL12p40 yellow, IL12p70 red, MCP1 grey, MIP1a orange, MIP1b black. (**R**) number of satellite cells, counted by cytometry in one specific Tibialis anterior in the control (non-injured), 18h, 1 month, 3 month and 28 days after re-injury. **p < 0.01; ***p < 0.001; no star, statistically non significant.
(TIF)

S10 Fig Summary of BaCl2 injury model regeneration process. (**A-D**) Haematoxylin and eosin stain 18h (**A**); 4 days, (**B**); 12 days (**C**) and 1 month (**D**) post-injury. Scale bar represent 50 μm (**E-H**) Immunohistochemistry of CD31 (red) and Laminin (green) in the BaCl_2_ injury model, 18h (**E**), 4 days (**F**), 12 days (**G**) and 1-month (**H**) post injury. Scale bar represent 50 μm (**I-L**) Images show blood vessel organisation in 3D 18h (**I**), 4 days (**J**), 12 days (**K**), 1-month (**L**) post-injury; arrows indicate anastomoses. Scale bar represent 10 μm. (**M-P**) Count of the number of inflammatory cells per section 18h, 4 days, 12 days and 1 month post-injury. (**M**), number of CD3+ cells; (**N**) number of B220+ cells; (**O**) number of Gr1+ cells; (**P**) number of F4/80+ cells. ***p < 0.001; no star, statistically non significant. (**Q**) Luminex (multiplex assay) measuring the levels of cytokines in pg/g in control, 18h, 4 days, 12 days and 1 month post injury. Selected cytokines are displayed (IL6 blue, IL10 green, IL12p40 yellow, IL12p70 red, MCP1 grey, MIP1a orange, MIP1b black. (**R**) Number of satellite cells, counted by cytometry in one specific Tibialis anterior in the control (non-injured), 18h, 1 month, 3 month and 28 days after re-injury. *p < 0.05, **p < 0.01; ***p < 0.001; no star, statistically non significant.
(TIF)

## Abbreviations

BaCl_2_: Barium Chloride
CTX: Cardiotoxin
EdU: 5-Ethynyl-2’-dEoxyuridine
FACS: Fluorescence-Activated Cell Sorting
FGF: Fibroblast Growth Factor
FI: Freeze Injury
GDF11: Growth differentiation factor 11
GFP: Green Fluorescent Protein
H&E: Haematoxylin and Eosin
IL: Interleukin
IM: Intra Muscular
LIF: Leukemia inhibitory factor
MCP-1: Monocyte Chimoattractant Protein-1
M-CSF: Macrophage Colony Stimulating Factor
MIG: Monokine induced by gamma interferon
MIP-2: Macrophage Inflammatory Protein 2
MP: Macrophage
NTX: Notexin
PBS: Phosphate Saline Buffer
PDGF-BB: Platelet-Derived Growth Factor BB
SC: Satellite cells
TA: *Tibialis Anterior*
VEGF: Vascular Endothelial Growth Factor
