# Supplementary figures and images for: Comparative Study of Injury Models for Studying Muscle Regeneration in Mice
Source: PLoS One. 2016 Jan 25;11(1):e0147198. doi: 10.1371/journal.pone.0147198 (PMC4726569; doi:10.1371/journal.pone.0147198)

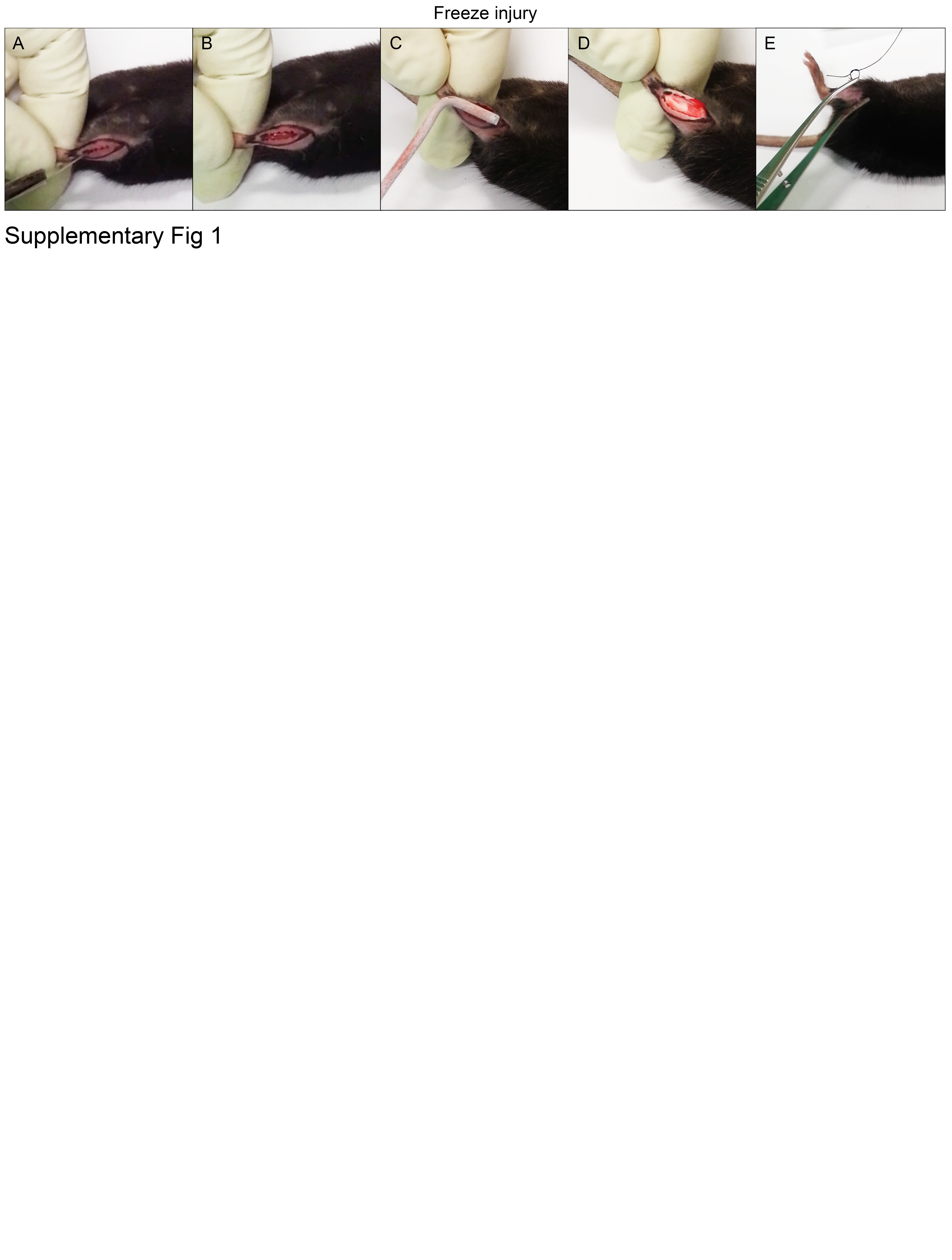

Supplement: S1 Fig — Freeze Injury: After skin incision (A) and muscle exposition (B) the Tibialis anterior was frozen with three consecutive cycles of freeze-thawing by applying for 15 sec a liquid nitrogen cooled metallic rod (C, D). The skin was then sutured (E). (TIF) [file pone.0147198.s001.tif]

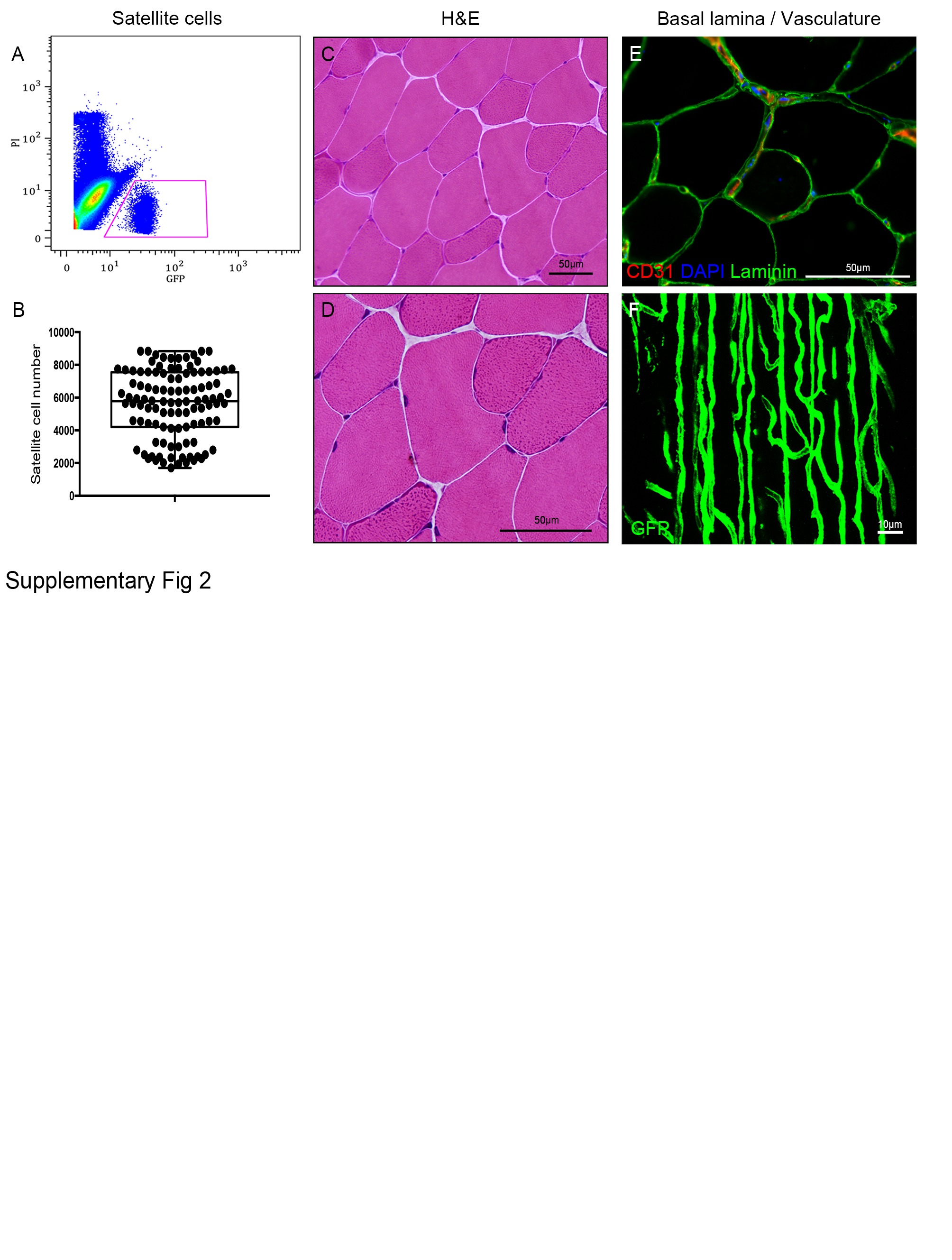

Supplement: S2 Fig — (A) Satellite cell counts by flow cytometery (Tg:Pax7nGFP mouse). GFP+ cells are the satellite cells; PI is propidium iodide to exclude dead cells. (B) Number of satellite cells in control Tibialis anterior muscle of n = 124 animals (ancillary and new data) by flow cytometry. (C, D) Haematoxylin and eosin stain of control, non-injured muscle. Scale bar represent 50 μm. (E) Control, uninjured TA muscle displaying vessel (CD31, red) and laminin (green) immunolabeling. Scale bar represent 50 μm. (F) Control uninjured TA muscle 3D imaging of microvascular network (Flk1GFP/+ mouse). Scale bar represent 10 μm. (TIF) [file pone.0147198.s002.tif]

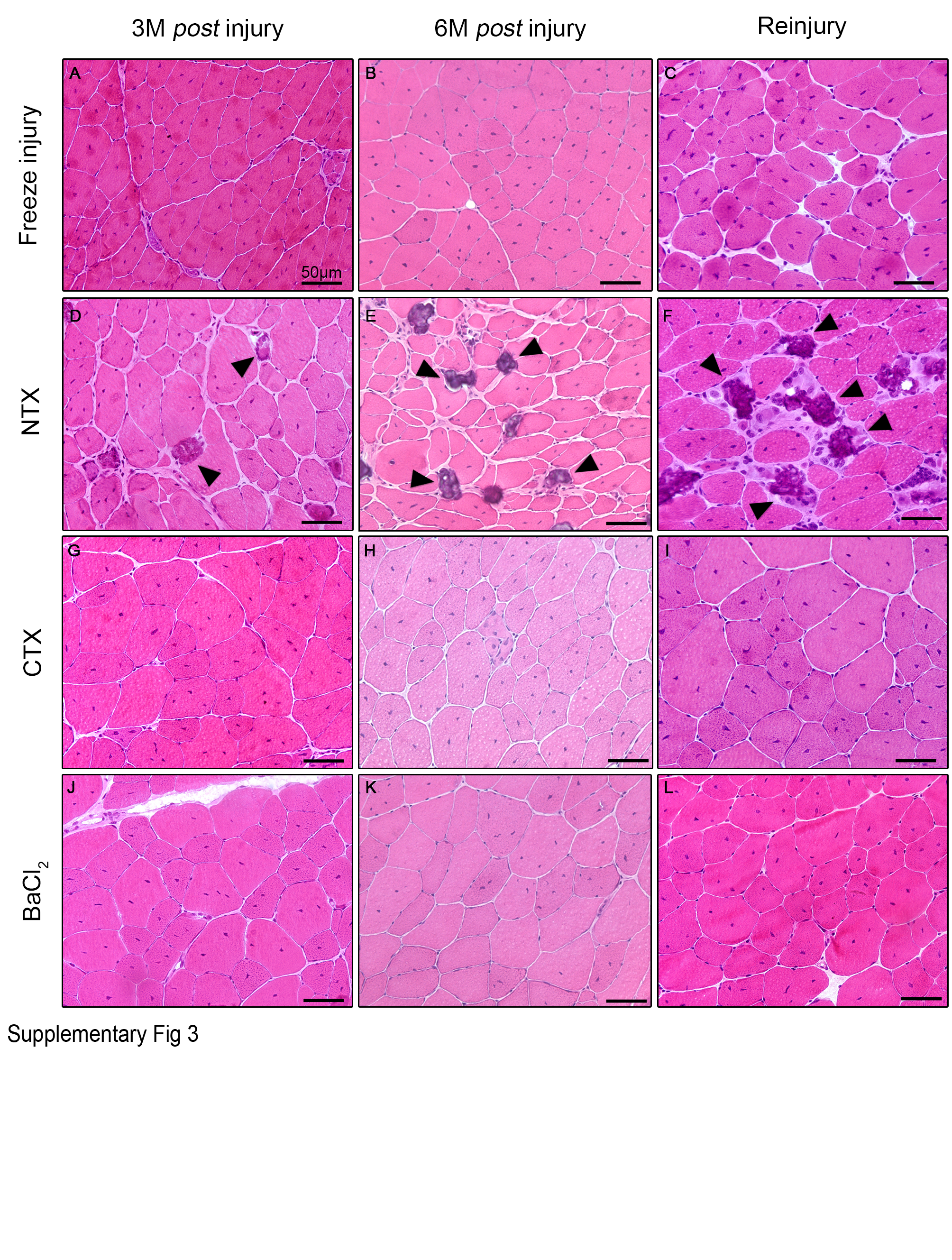

Supplement: S3 Fig — Haematoxylin and eosin staining on cryosections. (A) 3 months, (B) 6 months and (C) one month after freeze reinjury. (D) 3 months, (E) 6 months and (F) one month after NTX reinjury. (G) 3 months, (H) 6 months and (I) one month after CTX reinjury. (J) 3 months, (K) 6 months and (L) one month after BaCl2 reinjury. Scale bar represents 50 μm. (TIF) [file pone.0147198.s003.tif]

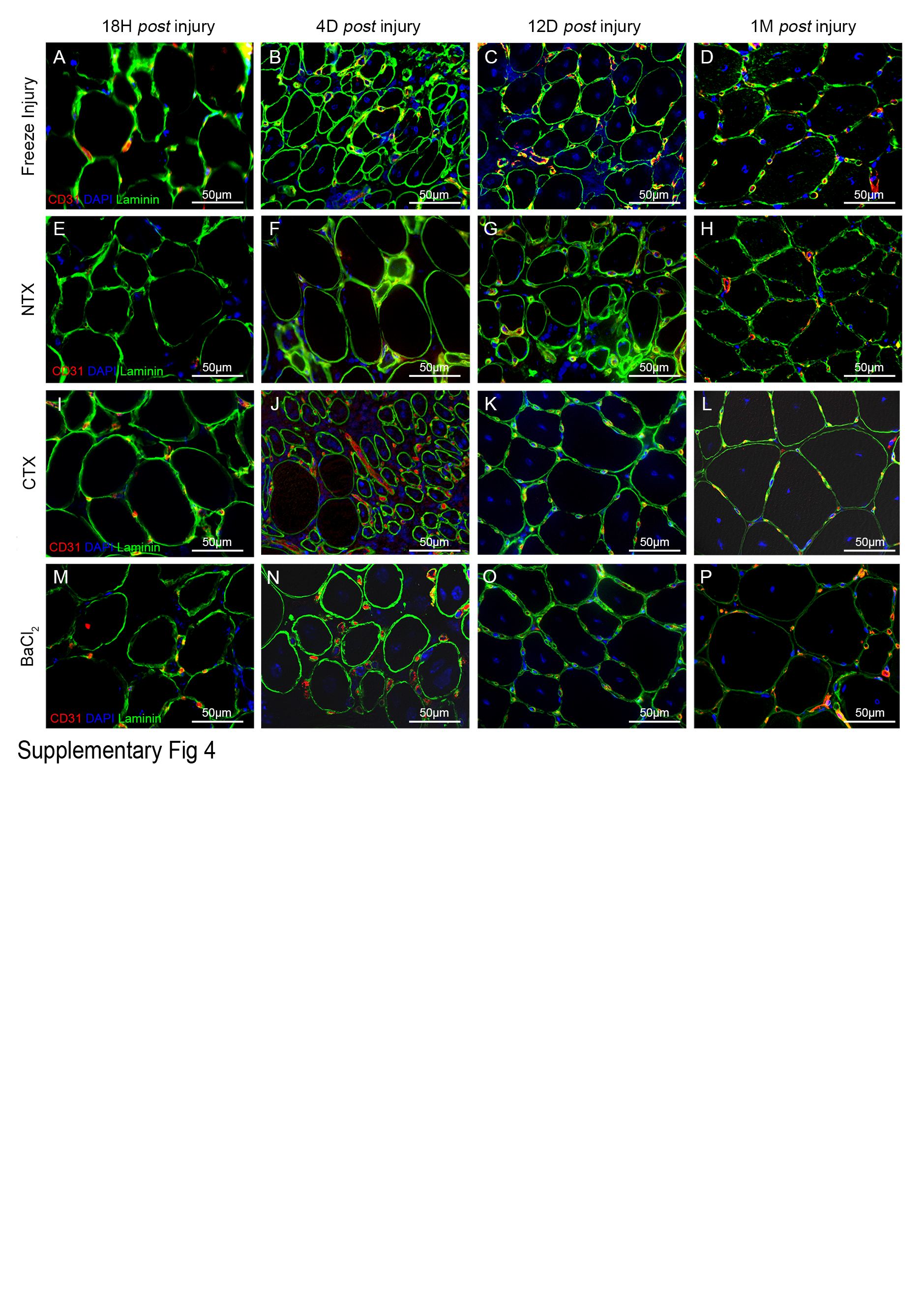

Supplement: S4 Fig — All images show blood vessel organisation in 2D (CD31 red / laminin green immunohistochemistry). (A) 18h, (B) 4 days, (C) 12 days and (D) one month post freeze injury. (E) 18h, (F) 4 days, (G) 12 days and (H) one month post NTX injury. (I) 18h, (J) 4 days, (K) 12 days and (L) one month post CTX injury. (M) 18h, (N) 4 days, (O) 12 days and (P) one month post BaCl2 injury. Scale bar represent 50 μm. (TIF) [file pone.0147198.s004.tif]

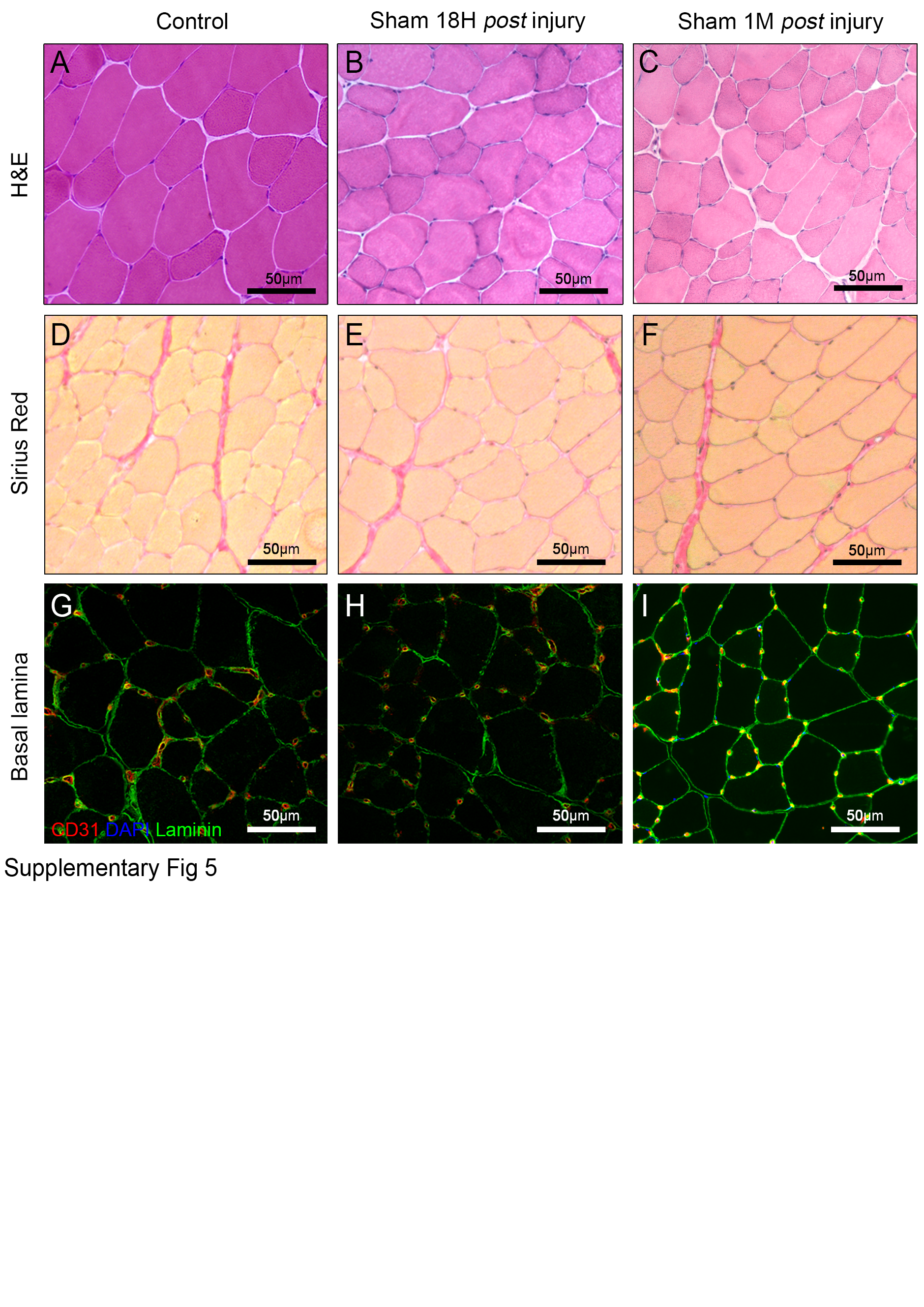

Supplement: S5 Fig — (A-C) Haematoxylin and eosin stain control (A), 18h (B), and 1 month (C), after aperture of the skin. (D-F) Sirius Red staining (collagen deposits) on control (D), 18h (E), and 1 month (F), post open skin. (G-I) Immunohistochemistry of CD31 (red) and Laminin (green) in the freeze injury model, control (G) 18h (H) and 1 month (I) after aperture of the skin. Scale bar represent 50 μm. (TIF) [file pone.0147198.s005.tif]

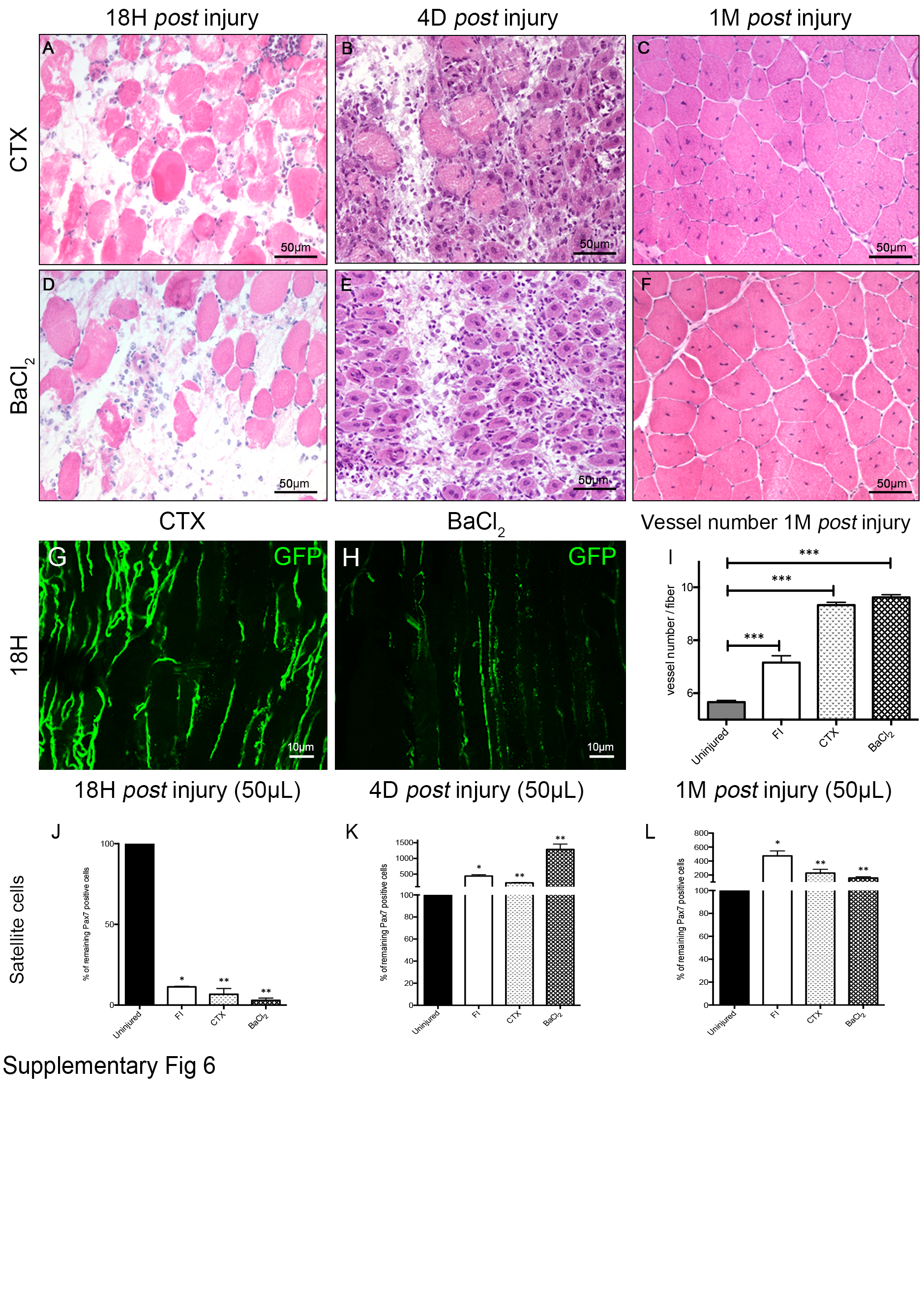

Supplement: S6 Fig — Haematoxylin and eosin staining on cryosections. (A) 18h, (B) 4 days and (C) one month after CTX injury. (D) 18h, (E) 4 days and (F) one month after BaCl2 injury. Scale bar represents 50 μm. Images show blood vessel organisation in 3D after z-stack reconstitutions of scanned sectioned TA from Flk1GFP/+ mouse. Vessel organisation after freeze injury 18h (H) and BaCl2 injury 18h (I). Scale bar represents 10 μm. (J) Vessel numbers per fibre 1 month after injury 50μL in all injury models. (K-M) Percentage of remaining Pax7 positive cells (K) 18h, (L) 4 days and (M) 1 month post-injury on TA sections. (N-P) Percentage of activated Ki67 positive satellite cells (N) 18h, (O) 4 days and (P) 1 month after injury. Data are represented as means±s.d. *p < 0.05; **p < 0.01; ***p < 0.001; no star, statistically non significant. (TIF) [file pone.0147198.s006.tif]

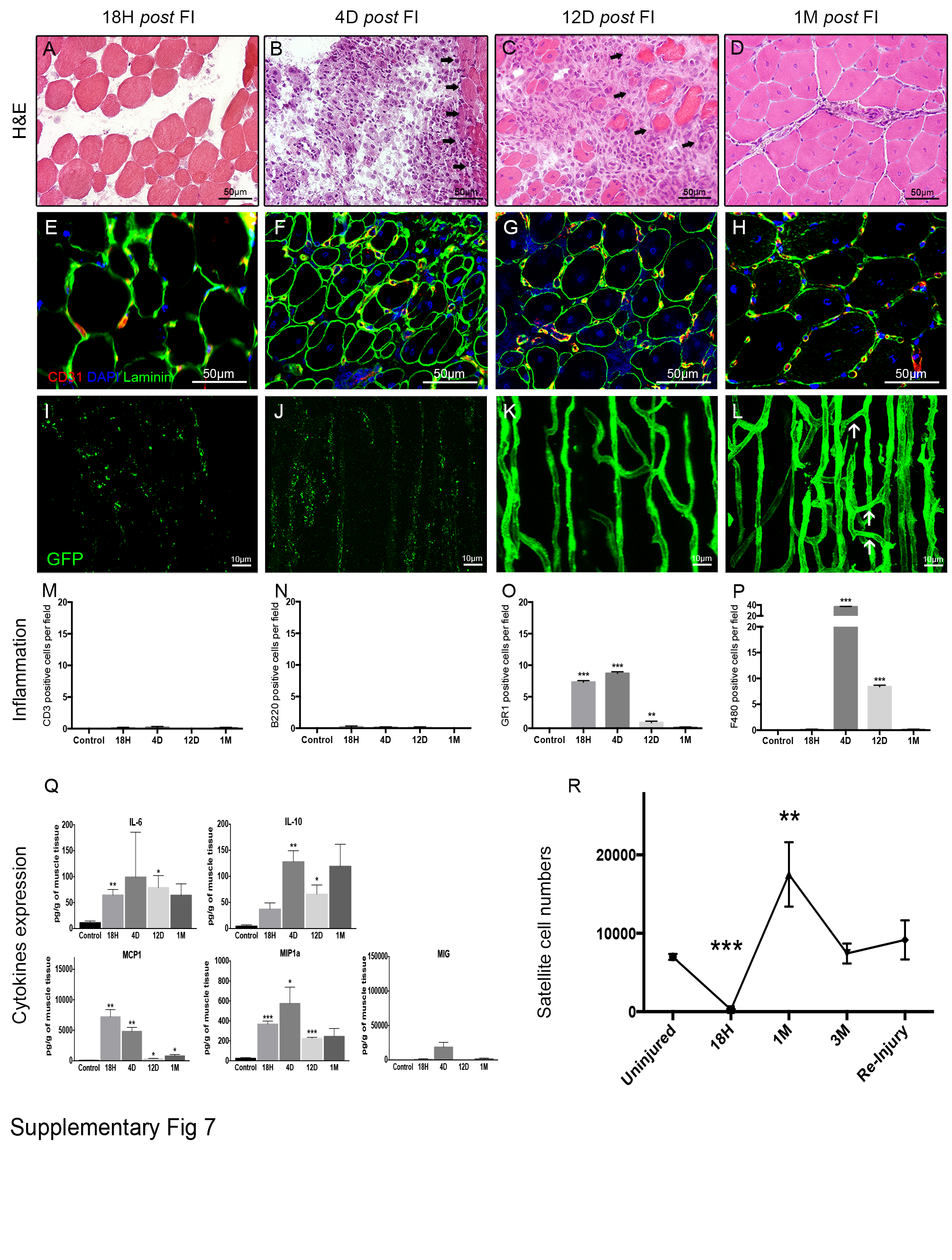

Supplement: S7 Fig — (A-D) Haematoxylin and eosin stain 18h (A); 4 days, arrows indicate regeneration front (B); 12 days arrows indicate regeneration front (C) and 1 month (D) post-injury. Scale bar represent 50 μm (E-H) Immunohistochemistry of CD31 (red) and Laminin (green) in the freeze injury model, 18h (E), 4 days (F), 12 days (G) and 1-month (H) post-injury. Scale bar represent 50 μm (I-L) Images show blood vessel organisation in 3D 18h (I), 4 days (J), 12 days (K), 1-month (L) post-injury. Scale bar represent 10 μm, arrows indicate anastomoses. (M-P) Count of the number of inflammatory cells per section 18h, 4 days, 12 days and 1 month post-injury. (M), number of CD3+ cells; (N) number of B220+ cells; (O) number of Gr1+ cells; (P) number of F4/80+ cells. ***P < 0.001; no star, statistically non significant. (Q) Luminex (multiplex assay) measuring the levels of cytokines in pg/g in control, 18h, 4 days, 12 days and 1 month post injury. Selected cytokines are displayed (IL6 blue, IL10 green, IL12p40 yellow, IL12p70 red, MCP1 grey, MIP1a orange, MIP1b black. (R) number of satellite cells, counted by cytometry in one specific TA muscle in the control (non-injured), 18h, 1 month, 3 month and 28 days after re-injury. **p < 0.01; ***p < 0.001; no star, statistically non significant. (TIF) [file pone.0147198.s007.tif]

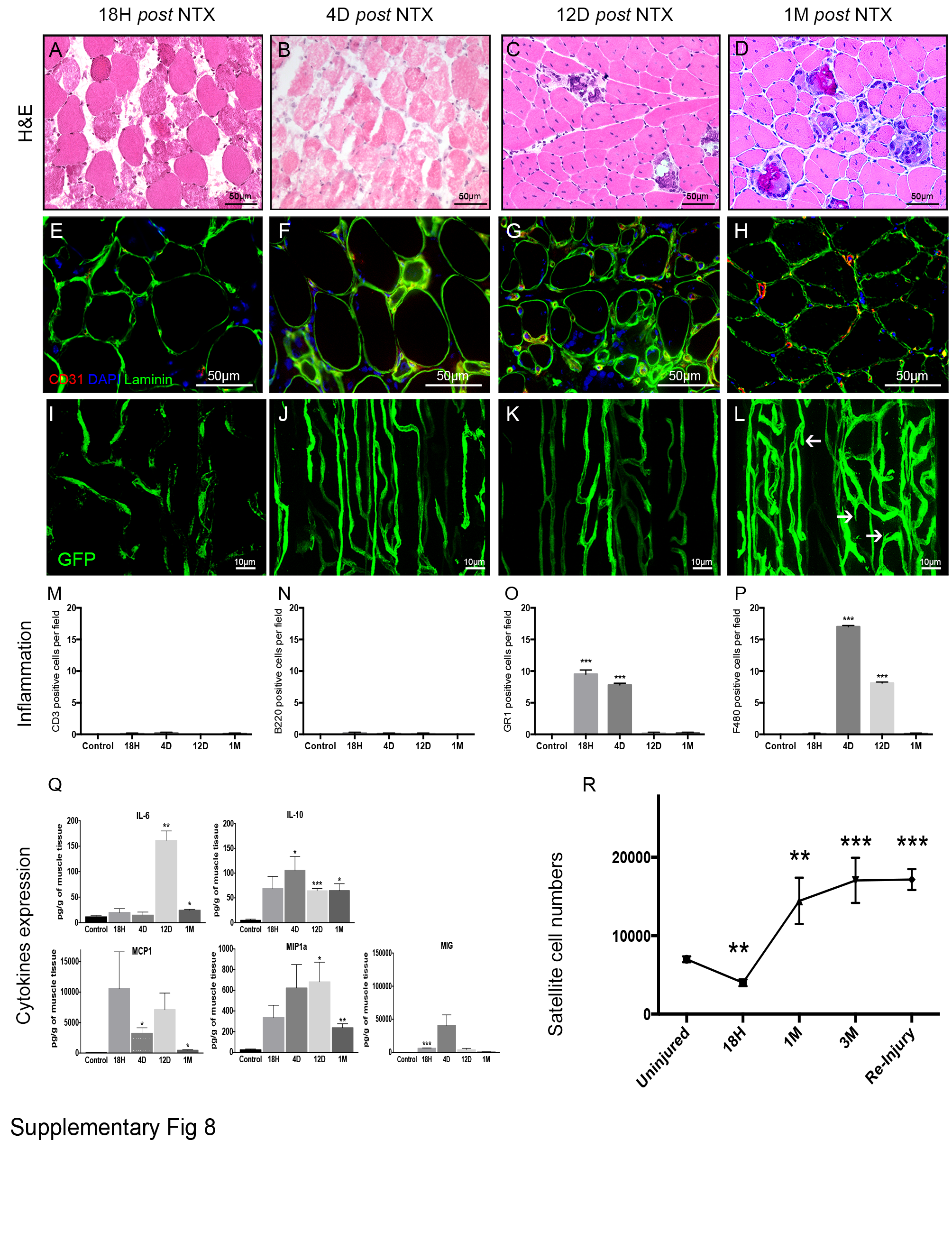

Supplement: S8 Fig — (A-D) Haematoxylin and eosin stain 18h (A); 4 days, (B); 12 days (C) and 1 month (D) post-injury. Scale bar represent 50 μm (E-H) Immunohistochemistry of CD31 (red) and Laminin (green) in the NTX injury model, 18h (E), 4 days (F), 12 days (G) and 1-month (H) post injury. Scale bar represent 50 μm (I-L) Images show blood vessel organisation in 3D 18h (I), 4 days (J), 12 days (K), 1-month (L) post-injury arrows indicate anastomoses. Scale bar represent 10 μm. (M-P) Count of the number of inflammatory cells per section 18h, 4 days, 12 days and 1 month post-injury. (M), number of CD3+ cells; (N) number of B220+ cells; (O) number of Gr1+ cells; (P) number of F4/80+ cells. ***p < 0.001; no star, statistically non significant. (Q) Luminex (multiplex assay) measuring the levels of cytokines in pg/g in control, 18h, 4 days, 12 days and 1 month post-injury. Selected cytokines are displayed (IL6 blue, IL10 green, IL12p40 yellow, IL12p70 red, MCP1 grey, MIP1a orange, MIP1b black. (R) number of satellite cells, counted by cytometry in one specific tibialis anterior muscle in the control (non-injured), 18h, 1 month, 3 month and 28 days after re-injury. **p < 0.01; ***p < 0.001; no star, statistically non significant. (TIF) [file pone.0147198.s008.tif]

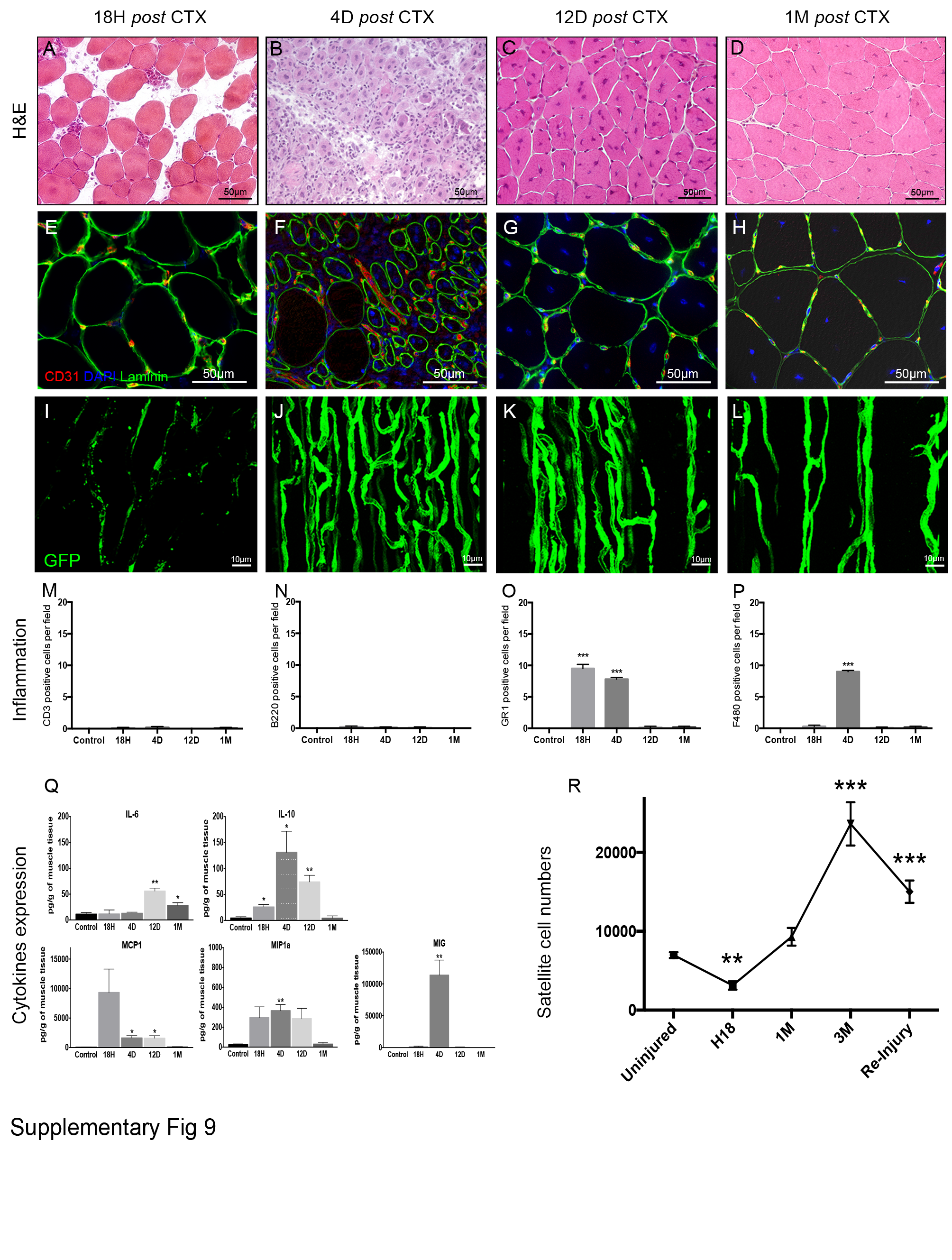

Supplement: S9 Fig — (A-D) Haematoxylin and eosin stain 18h (A); 4 days, (B); 12 days (C) and 1 month (D) post-injury. Scale bar represent 50 μm (E-H) Immunohistochemistry of CD31 (red) and Laminin (green) in the CTX injury paradigm, 18h (E), 4 days (F), 12 days (G) and 1-month (H) post-injury. Scale bar represent 50 μm (I-L) Images show blood vessel organisation in 3D 18h (I), 4 days (J), 12 days (K), 1-month (L) post-injury; arrows indicate anastomoses. Scale bar represent 10 μm. (M-P) Count of the number of inflammatory cells per section 18h, 4 days, 12 days and 1 month post-injury. (M), number of CD3+ cells; (N) number of B220+ cells; (O) number of Gr1+ cells; (P) number of F4/80+ cells. ***p < 0.001; no star, statistically non significant. (Q) Luminex (multiplex assay) measuring the levels of cytokines in pg/g in control, 18h, 4 days, 12 days and 1 month post injury. Selected cytokines are displayed (IL6 blue, IL10 green, IL12p40 yellow, IL12p70 red, MCP1 grey, MIP1a orange, MIP1b black. (R) number of satellite cells, counted by cytometry in one specific Tibialis anterior in the control (non-injured), 18h, 1 month, 3 month and 28 days after re-injury. **p < 0.01; ***p < 0.001; no star, statistically non significant. (TIF) [file pone.0147198.s009.tif]

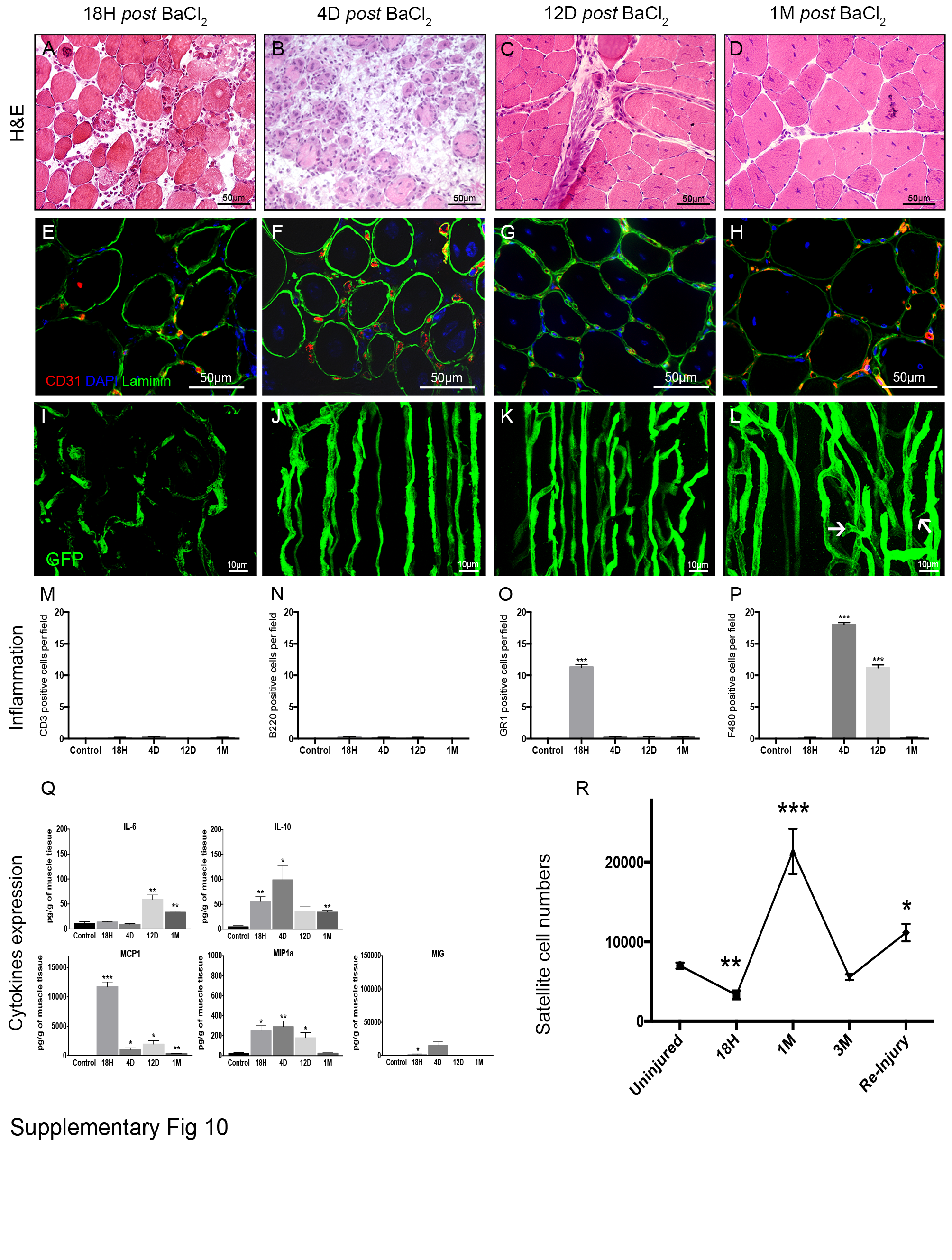

Supplement: S10 Fig — (A-D) Haematoxylin and eosin stain 18h (A); 4 days, (B); 12 days (C) and 1 month (D) post-injury. Scale bar represent 50 μm (E-H) Immunohistochemistry of CD31 (red) and Laminin (green) in the BaCl2 injury model, 18h (E), 4 days (F), 12 days (G) and 1-month (H) post injury. Scale bar represent 50 μm (I-L) Images show blood vessel organisation in 3D 18h (I), 4 days (J), 12 days (K), 1-month (L) post-injury; arrows indicate anastomoses. Scale bar represent 10 μm. (M-P) Count of the number of inflammatory cells per section 18h, 4 days, 12 days and 1 month post-injury. (M), number of CD3+ cells; (N) number of B220+ cells; (O) number of Gr1+ cells; (P) number of F4/80+ cells. ***p < 0.001; no star, statistically non significant. (Q) Luminex (multiplex assay) measuring the levels of cytokines in pg/g in control, 18h, 4 days, 12 days and 1 month post injury. Selected cytokines are displayed (IL6 blue, IL10 green, IL12p40 yellow, IL12p70 red, MCP1 grey, MIP1a orange, MIP1b black. (R) Number of satellite cells, counted by cytometry in one specific Tibialis anterior in the control (non-injured), 18h, 1 month, 3 month and 28 days after re-injury. *p < 0.05, **p < 0.01; ***p < 0.001; no star, statistically non significant. (TIF) [file pone.0147198.s010.tif]
